# Nutrient intakes of pregnant and lactating women in Indonesia and Malaysia: Systematic review and meta-analysis

**DOI:** 10.3389/fnut.2023.1030343

**Published:** 2023-03-30

**Authors:** Rina Agustina, Davrina Rianda, Wanda Lasepa, Fitya S. Birahmatika, Vladimir Stajic, Rachmi Mufida

**Affiliations:** ^1^Department of Nutrition, Faculty of Medicine, Universitas Indonesia - Dr. Cipto Mangunkusumo General Hospital, Jakarta, Indonesia; ^2^Human Nutrition Research Center, Indonesian Medical Education, and Research Center (HNRC-IMERI), Faculty of Medicine, Universitas Indonesia, Jakarta, Indonesia; ^3^Blackmores Institute, Blackmores Limited, Sydney, NSW, Australia

**Keywords:** Indonesia, Malaysia, pregnant women, lactating women, nutrient intake

## Abstract

**Introduction:**

Optimizing dietary intake during pregnancy and lactation is crucial to the growth and development of children and their lifelong health. We performed a systematic review and meta-analysis to describe the nutrient intakes of pregnant and lactating women in Indonesia and Malaysia, countries that are experiencing rapid nutrition transition in Southeast Asia.

**Methods:**

We screened 2,258 studies published between January 1980 and March 2021. The nutrient intakes of pregnant and lactating women were quantitatively analyzed to calculate the percentage of adequacy using national recommended daily allowances or nutrient intakes (RDA/RNI) and estimated average requirements (EAR) for micronutrients. Standardized mean differences (SMD) between dietary intake and RDA/RNI were determined for selected nutrients.

**Results:**

Fifty-three studies were included and showed that energy and macronutrient intakes among pregnant and lactating women in both countries were below the RDA/RNI. In addition, most studies reported that women failed to meet the EAR for vitamin D (<70% EAR), vitamin E (<50% EAR), and water-soluble vitamins (<80% EAR) except for vitamin C and A among Malaysians. Moreover, calcium, potassium, and iron intakes of pregnant women were <60% EAR in Indonesia and <80% EAR in Malaysia. Phosphorus intake among pregnant women in both countries and sodium intake among Malaysian pregnant women exceeded 100% EAR. Indonesian lactating women had <60% EAR for calcium and potassium intakes, while Malaysian women had more than 100% EAR. For 21 studies reporting overall protein intakes, the standard mean difference (SMD) was −2.26 (95% CI; −2.98, −1.54) below the RDA/RNI for pregnant women and −0.67 SMD (95% CI −15.7, −11.5) for lactating women. When the four studies from Malaysia were analyzed separately, protein intakes of pregnant women were above the RNI. Moreover, low intakes of vitamin D, vitamin C, calcium, and iron, but sufficient intakes in vitamin A, zinc, and phosphorus were observed in pregnant women in both countries.

**Conclusion:**

Dietary intakes of energy, macronutrients, and micronutrients (vitamin D, vitamin E, water-soluble vitamins, calcium, and iron) of pregnant and lactating women in Indonesia and Malaysia were below the recommendations. Important heterogeneities were observed even between these two countries for specific essential nutrient intakes. Innovative research and targeted programs to address specific deficiencies should be prioritized.

**Systematic review registration:**

https://www.crd.york.ac.uk/prospero/display_record.php?ID=CRD42021252204, identifier: CRD42021252204.

## Introduction

The first 1,000 days of life, spanning from conception to the age of two, is pivotal to the offspring's neurodevelopment and lifelong health. Adequate nutrient intake during pregnancy and lactation has been associated with maternal and child health outcomes and will ensure healthy growth and development of the children (1). Poor nutrient intake has been associated with poor maternal and infant outcomes such as preeclampsia, low birth weight, as well as increased risks of maternal and infant mortality (2).

Chronic energy deficiency (CED) commonly occurs among pregnant women and women of reproductive age in developing countries due to multiple factors, such as low socioeconomic status, living in rural areas, family size, inadequate meal frequency, and low dietary quality (3, 4). CED is one of the major contributors to maternal anemia, resulting in a high risk of prematurity and low birth weight (5, 6). In addition, several studies showed that the pregnant women had low energy intake, anemia, and multi-micronutrient deficiencies (e.g., iron, folic acid, vitamin A, vitamin D, vitamin B12, zinc, and iodine) (7, 8). For instance, inadequate intake of iron during pregnancy can cause low birth weight, premature delivery, and impaired cognitive development of infants. Similarly, poor folic acid intake during pregnancy may lead to adverse birth outcomes (e.g., neural tube defects) and an increased risk of rising homocysteine level of the mothers, preeclampsia, and preterm delivery (9, 10).

Meanwhile, micronutrient supplement consumption was not a popular practice among pregnant women in Indonesia and Malaysia, particularly for certain nutrients such as vitamin D (11, 12). Moreover, supplementation programs for pregnant and postpartum women might not be implemented evenly across countries. Overall, the dietary pattern among pregnant women showed a tendency to have a healthier diet than before pregnancy with more consumption of vegetables and protein-rich foods in the early pregnancy (13). Yet, it seemed the adherence did not last until the last trimester. A few studies reported inadequacy of micronutrient intake, particularly iron and zinc, among lactating women in Indonesia and Malaysia that may impact inadequacy of these nutrients to breastfed children (14).

Asia is a continent of diverse cultures, represented by various traditional foods and culinary practices. Various traditional dishes are healthy and promote fruits and vegetable consumptions. People residing in developing countries of Southeast Asia region inevitably experienced diet transition that shifted food consumption to more practical and modernized yet heavily processed foods, neglecting traditional foods that are healthy and rich in bio-active compounds (15). Moreover, ultra-processed food (UPF) is also rapidly growing in Indonesia and Malaysia. Although dietary diversity in Indonesia was increasing, the shares of prepared foods were also escalating. Living in urban areas, particularly in the capital city, contributed to less intake of traditional diets; which included lower expenditures for rice and higher expenditures for prepared foods (16). A recent study in Malaysia revealed that energy supply remained excessive, surpassing the average calorie requirement. Along with white rice as the main staple food, table sugar was the most widely consumed food in Malaysia. This dietary trend should be alarming for the country considering the rise of obesity prevalence and non-communicable diseases, such as hypertension, diabetes mellitus, and hypercholesterolemia in this country (17). De Nucci et al. reported an increase in UPF consumption globally, namely sweets, salty snacks, and packaged bread, despite a decline in other UPF groups (i.e., ready-to eat and delivery foods, sugary drinks) particularly during the COVID-19 pandemic (18).

A balanced diet with appropriate nutrient intake is recommended during pregnancy and lactation. However, previous studies highlighted that poor diet quality was predominant among healthy (19) and pregnant women in Indonesia (20). A similar dietary pattern among indigenous (21) and pregnant women (22) in Malaysia was also reported. Some studies showed that lactating women in Southeast Asian countries preferably consumed rice-based and high-fat diets to fulfil the calorie intake than adding protein source foods in their diets. This habit may lead the lactating women in this region to have low dietary quality and diversity, low protein consumption as well as low consumption of fruits and vegetables. This trend is also observed among European and Urban Chinese lactating women (23, 24). This trend of dietary pattern may affect the nutrient adequacy of pregnant and lactating women and will further influence maternal and child nutrition and health outcomes. Therefore, this review aimed to provide an overview of nutrient intake adequacy among pregnant and lactating women in Indonesia and Malaysia, countries that are experiencing rapid nutrition transition in Southeast Asia. Findings of this review can be used to provide information and insight for planning the future strategy on nutrition and health intervention to prevent nutrient intake inadequacy among pregnant and lactating women in developing countries.

## Materials and methods

### Search strategy

The literature on nutrient intake in Indonesia and Malaysia was systematically searched through four electronic databases: PubMed, Scopus, ProQuest, and Cochrane. A manual search was conducted when certain nutrient intakes were not found in the directories. The search strategy was designed to find published studies in English or Indonesian language. The search terms used are shown in the Supplementary Table 1. Preferred Reporting Items for Systematic reviews and Meta-Analysis (PRISMA) were used as a guideline for this systematic review. The protocol was submitted to the International Prospective Register of Systematic Reviews (PROSPERO) database (registration number CRD42021252204).

### Eligibility criteria

Nutrient intake and supplement consumption were determined as the outcomes during the selection of studies. Inclusion criteria of the study were published papers between the period of January 1980 and March 2021 that involved healthy and unhealthy pregnant and lactating women (mothers who breastfed children up to 2 years of age), assessed the quantity of dietary intake (i.e., kcal in energy; gr in macronutrient, etc.) using nutritional assessment methods that can quantify actual and habitual nutrient intake [24-h food recall, food record, and semi quantitative-food frequency questionnaire (SQ-FFQ)]. The exclusion criteria were applied to studies that had no abstract, were not in English or Indonesian language, had irrelevant topic, and were not human studies. Moreover, intervention studies were included if the baseline data were provided.

### Data collection

**Figure 1:**
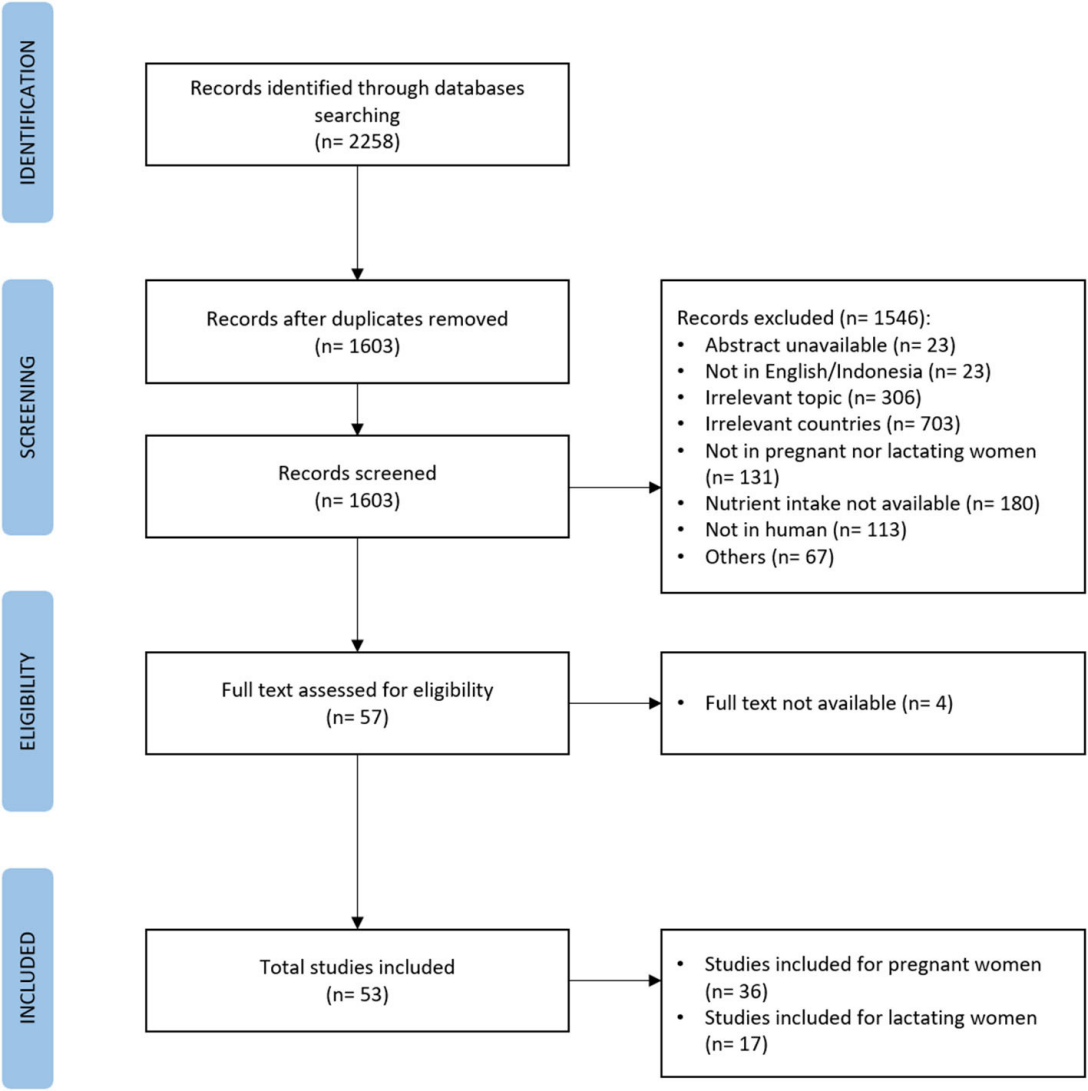
Prisma flow diagram of systematic review.

The titles and abstracts were screened by two reviewers (FS and RM). Any disagreements over the included studies were resolved through discussions. The full text was assessed based on the exclusion criteria after the duplicate studies were removed. The flow diagram of studies using PRISMA guidelines is provided in Figure 1.

Extracted information of the eligible studies was summarized in Table 1 which describes the country, first author, year of publication, type of study, study population characteristics, dietary assessment method, nutrient assessment, and main findings. The analysis was performed for all studies by calculating the percentage of adequacy using national recommended daily allowances or nutrient intakes (RDA/RNI) and estimated average requirements (EAR) for micronutrients.

**Table 1:** Studies characteristics of pregnant and lactating women in Indonesia and Malaysia.

**References, country**	**Study design**	**Subject characteristics (age of pregnancy/breastfeeding status/living area/subjects)**	**Dietary assessment method; sources of nutrient**	**Nutrient intake identified**	**Intake (%RDA, %RNI, %EAR)**
Aji et al. (25), Indonesia (West Sumatera)	Cross-sectional	Pregnant women; <13 weeks; urban and rural; 203 subjects	FFQ; foods only	Vitamin D	7.9 mcg (52.8, 79.2)
Aji et al. (26), Indonesia (West Sumatera)	Cross-sectional	Pregnant women; <13 weeks; urban and rural; 232 subjects	SQ-FFQ; foods and supplement	Vitamin D	5.3 mcg (35.5, 53.2)
Aji et al. (12), Indonesia (West Sumatera)	Cross-sectional	Pregnant women; above 28 weeks; coastal and mountainous area; 203 subjects	SQ-FFQ; foods and supplement	Energy	2,444 kcal (95.8, –)
				Carbohydrate	267 g (66.7, –)
				Protein	106.3 g (118.1, –)
				Fat	109.6 g (162.8, –)
				Calcium	785 mg (88–92, 115–119)
				Vitamin D	7.9 mcg (52.8, 79.2)
Angkasa et al. (27), Indonesia (Jakarta)	Longitudinal study	Pregnant women; above 32 weeks; urban area; 315 subjects	SQ-FFQ; foods only	Energy	1,846 kcal (76.7, –)
				Fat	63.4 g (94.2, –)
				Omega-3	1.3 g (96.4, –)
				ALA	1.1 g (75.7, –)
				DHA	0.17 g (85, –)^a^
				EPA	0.12 g (120, –)^b^
Angkasa et al. (28), Indonesia (Jakarta)	Cross-sectional (validation study)	Pregnant women; above 32 weeks; urban area; 100 subjects	FFQ; foods only	Energy	2,025 kcal (79.4, –)
				Fat	64.2 g (95.4, –)
				Total omega-3	1.3 g (96.4, –)
				Omega-6	1.3 g (9.6, –)
				EPA	0.14 g (140, –)
				ALA	1.1 g (75.7)
				DHA	0.17 g (85, –)
Basir et al. (29, 30), Malaysia (Pahang)	Longitudinal study	Exclusive breastfeeding: a term singleton babies; urban area; 32 subjects	3 days dietary recall; foods and supplement	Energy	1,479–2,022 kcal (61.6– 84.3, –)
				Protein	58.3–72 g (80.9–100, –)
				Carbohydrate	188.8–272 g (48.4–69.7, –)
				Fat	33.4–56.9 g (50.6–86.2, –)
				Saturated Fat	2.9–12.4 g (11–47.1, –)
Bukhary et al. (31), Malaysia (Petaling)	Cross-sectional	Pregnant women; first trimester; urban area; 396 subjects	FFQ; foods and supplement	Vitamin D	5.1 mcg (33.7, 51)
Daniels et al. (14), Indonesia (Sumedang)	Cross sectional	Exclusive breastfeeding; rural area; 110 subjects	12-h daytime weighed food intakes + 12-h maternal recall; foods only	Energy	2,211 kcal (85.7, –)
				Calcium	613 g (51, 61)
				Cobalamin	2.5 mcg (50, 60)
				Iron	18.3 mg (101.6, 122)
				Niacin	12.8 mg (75.3, 97.9)
				Potassium	1.1 g (21.5, 39.2)
				Riboflavin	1.7 mg (106.3, 127.5)
				Thiamin	1.4 mg (93.3, 112)
				Vitamin A	501 mg RE (52.7, 73.8)
				Pyridoxine	1.3 mg (68.4, 82.1)
				Zinc	12.8 mg (98.4, 118.2)
Wijaya-Erhardt et al. (32), Indonesia (Central Java)	Randomized intervention trial	Pregnant women; 12–20 weeks; rural area; 108 subjects (intervention), 114 subjects (control)	Single 24-h dietary recall; foods only	Energy	1,166–1,246 kcal (45.7–62.7, –)
				Protein	33.3–36.6 g (47.5–52.2, –)
				Fat	30.6–31.7 g (45.4–47.1, –)
				Iron	6.82–7.65 mg (25.−51, 30.3–61.2)
				Vitamin C	47.7–50.3 mg (56.1–59.2, 67.3–71.0)
Fikawati et al. (33), Indonesia	Cross-sectional	Vegetarian and non-vegetarian lactating women; a term singleton delivery; urban area; 33 subjects	SQ-FFQ; foods only	Energy	Vegetarian: 2,361 kcal (89.1, –), non-vegetarian: 1,855 kcal (70, –)
Fikawati and Syafiq (34, 35), Indonesia (Depok)	Longitudinal study	Exclusive and non-exclusive breastfeeding; urban area; 44 subjects	Single 24-h recall; foods only	Energy	Exclusive breastfeeding: 1,482 kcal (55.9, –), non-exclusive breastfeeding: 1,887 kcal (71.2, –)
Gibson et al. (36), Indonesia (Bandung and Sumedang)	Longitudinal study	Lactating women; urban and rural area; 212 subjects	Food record; foods and supplement	Calcium	471–497 mg (39–41, 47–50)
				Iron	12–12.1 mg (65.5–67, 78.6–81.3)
				Niacin	10.5–10.9 mg (61.8–64.1, 80.3–83.4)
				Potassium	0.9–1 g (17.6–19.6, 32–35.7)
				Riboflavin	1.2 mg (75, 90)
				Thiamin	1–1.1 mg (66.7–73.3, 80–88)
				Vitamin A	387–398 mcg RE (40.7–41.9, 57–58.7)
				Pyridoxine	1.1–1.3 mg (57.9–68.4, 69.5–82.1)
				Cobalamin	2.1–2.3 mg (42–46,50.4–55.2)
				Zinc	9.3–9.5 mg (71.5–73, 85.8–87.6)
Sawal Hamid et al. (37), Malaysia (Selangor)	Cross-sectional	Pregnant women; above 6 weeks; urban area; 78 subjects	Single 24-h dietary recall; foods and supplement	Energy	2,310 kcal (109, –)
				Fiber	13 g (43.3, –)
				Vitamin A	1,189 mcg RE (148.6, 208.1)
				Vitamin C	125 mg (156.3, 187.5)
				Thiamine	20.2 mg (1,443, 1,731.4)
				Riboflavin	1.6 mg (114.3, 137.1)
				Niacin	1.1 mg (6.1, 7.9)
				Iron	23 mg (85.1, 102.2)
				Calcium	689 mg (69, 83)
				Phosphorus	1,201 mg (172, 207)
				Sodium	2,813 mg (187.5, 187.5)
				Potassium	2.5 g (53.1, 53.1)
Hartini et al. (38), Indonesia (Purworejo)	Longitudinal study	Pregnant women; rural area; all trimester; 450 subjects	Single 24-h dietary recall; foods only	Protein	41–52 g (58.6–74.3, –)
				Fat	37–51 g (55–75.8, –)
				Carbohydrate	273–357 g (68.3–89.3, –)
				Vitamin A	249.6–716.7 mcg RE (27.7–134.3, 38.8–111.5)
				Calcium	276–443 mg (23–96.6, 27.6–125.4)
				Iron	12–17 mg (44.44–113.3, 53.33–136)
Hartriyanti et al. (35), Indonesia	Review	Pregnant women in Indonesia (West Java, Yogyakarta, Central Java, East Java, Papua): 7 studies	Semi-quantitative food frequency questionnaire; 24-h dietary recall (single and multiple)	Energy	1,591–2,239 kcal (65–91.4, –)
				Protein	42.5–58.8 g (60.7–84, –)
				Fat	46.1 g (68.5, –)
				Carbohydrate	248.5 g (62.1, –)
				Vitamin A	992.5 mcg (110.3, 154.4)
				Vitamin C	68.1–145.9 mg (80.1–171.6, 96.1–205.9)
				Iron	9.6–30.4 mg (35.5–112.5, 42.6–135.1)
				Zinc	12.9 mg (107.5, 129)
				Calcium	360–614 mg (30–51, 36–61)
				Sodium	2,356 mg (157.08, 157.1)
				Potassium	1,911 mg (40.6, 40.6)
Hasbullah et al. (39), Malaysia (Seremban)	Cohort	Pregnant women; above 10 weeks; urban area; 294 subjects	FFQ; foods only	Energy	1,980 kcal (90.8, –)
				Carbohydrate	290 g (81.9, –)
				Protein	76.3 g (97.8, –)
				Fat	56 g (71.8, –)
				Fiber	14.8 g (49.3, –)
				Calcium	740 mg (74, 62)
				Iron	16.3 mg (60.3, 72.4)
Hassan et al. (40), Malaysia (Sepang)	Quasi-experimental	Pregnant women; below 24 weeks of gestation; 162 subjects (intervention), 162 subjects (control)	Weighed food records in 3 days (2 weekdays and 1 weekend); foods and supplement	Iron	23.0–24.8 mg (85–91.8, 102–110.2)
Ilmiawati et al. (41), Indonesia (Padang)	Cross-sectional	Pregnant women; third trimester; urban area; 88 subjects	Single 24-h dietary recall; foods only	Vitamin D	5.6 mcg/1,000 kcal/day
Kamaruzzaman et al. (42), Malaysia (Kuantan)	Cross-sectional	Exclusive breastfeeding; urban area; 70 subjects	3 days food record; foods only	Protein	85.7 g (119, –)
				Carbohydrate	259.1 g (66.4, –)
				Fat	64.2 g (81.3, –)
Kardjati et al. (43), Indonesia (Madura)	Randomized control trial (RCT)	Pregnant women; 26–28 weeks of gestation; rural area; 360 subjects	3-days food weighing; foods and supplement	Energy	1,500 kcal (58.8, –)
				Protein	41 g (58.6, –)
				Fat	18 g (26.7, –)
				Retinol	443 mcg (49.2, 68.9)
				Thiamin	0.8 mg (59.3, 71.1)
				Riboflavin	0.4 mg (25.7, 30.9)
				Iron	6.7 mg (24.8–44.7, 29.7–53.6)
				Calcium	167 mg (14, 17)
Kardjati et al. (44), Indonesia (Madura)	Randomized trial	Pregnant women; all trimester; rural area; 4,376 subjects	Weighed food records in 3 consecutive days; foods	Energy	1,532–1,654 kcal (63–64.9, –)
				Protein	40–43 g (45.6–65.6, –)
Khor et al. (45), Malaysia (Kuala Lumpur)	Cross-sectional	Lactating women; 2–16 week postpartum term singleton delivery; urban area; 20 subjects	24-h dietary recall (3 days); foods only	Energy	2,271–2,436 kcal (94.6–101.5, –)
				Protein	83.6–100.8 gr (117.7–141.9, –)
				Fat	64.6–77.1 g (81.8–116.8, –)
				Carbohydrate	338.2–368.2 g (86.7–122.7, –)
				SFA	16.2–17.3 g (60.7–64.8, –)
Kneebone et al. (46), Malaysia (Penang)	Cross-sectional	Lactating women; all trimester; urban area; 4 subjects	FFQ and single 24-h dietary recall; foods only	Energy	1,243 kcal (53.1, –)
				Carbohydrate	139.8 g (36.8, –)
				Protein	37.3 g (51.8, –)
				Fat	45.6 g (57.7, –)
Lee et al. (47), Malaysia (Selangor)	Cross-sectional	Pregnant women; over 37 weeks; urban area; 248 subjects	SQ-FFQ; foods only	Vitamin D	8.3 mcg (55.3, 83)
Loy et al. (48), Malaysia (Kelantan)	Cross-sectional	Pregnant women; 28–38 weeks; urban area; 121 subjects	SQ-FFQ; foods only	Energy	2,042 kcal (86.2, –)
				Protein	83.9 g (108,–)
				Carbohydrate	306.4 g (79.6, –)
				Fat	54.6 g (70, –)
				Fiber	7 g (23.3)
				Sodium	2,737 mg (182, 182)
				Potassium	1,648 mg (35.1, 35.1)
				Calcium	821 mg (82, 98)
				Iron	20,1 mg (74.4, 89.3)
				Phosphorus	998 mg (143, 172)
				Vitamin A	959 mcg (1,199, 167.8)
				Vitamin C	121.2 mg (151.5, 181.8)
				Thiamin	1.6 mg (114.3, 137.1)
				Riboflavin	2 mg (142.9, 171.4)
				Niacin	15.9 mg (88.3, 114.8)
Loy et al. (49), Malaysia (Kelantan)	Validation study	Pregnant women; 12–22 weeks and 28–38 weeks; urban area; 177 subjects	SQ-FFQ and repeated 24-h dietary recall; foods only	Energy	1,811–2,021 kcal (78.4–87.5, –)
				Protein	69.9–78.9 g (90.8–101.2, –)
				Carbohydrate	245.6–306.4 kcal (63.8–81.6, –)
				Fiber	5.5–7 g (18.3–23.3, –)
				Fat	53.9 −56.5 g (69.1–72.4, –)
				Calcium	626–831 mg (63–83, 75–100)
				Iron	20.3–20.8 mg (69.6–75.1, 83.5–90.2)
				Niacin	12.8–15.9 mg (71.1–83.3, 92.4–114.8)
				Phosphorus	967–1,103 mg (138–158, 167–190)
				Potassium	1,552–1,647 mg (33–35, 33–35)
				Riboflavin	1.6–2 mg (114.3–142.9, 137.1–171.4)
				Sodium	2,123–2,586 mg (141.5–172.4, 141.5–172.4)
				Thiamin	1.1–1.6 mg (78.6–114.3, 94.3–137.1)
				Vitamin A	971.8–987.7 mcg, RE (121.5–123.5, 170.1–172.8)
				Vitamin C	96.4–127.2 mg (120.5–159, 144.6–190.8)
Loy et al. (50), Malaysia (Kelantan)	Cross-sectional	Pregnant women; ≥28 weeks; Hospital University Sains Malaysia; 108 subjects	SQ-FFQ; foods only	Energy	2,031 kcal (87.9, –)
Madanijah et al. (51), Indonesia (Bogor)	Cross-sectional	Pregnant women; 13 and < 28 weeks; rural area; 203 subjects	FFQ; foods only	Energy	1,447–1,678 kcal (56.7 −65.8, –)
				Protein	37–50 g (52.9–71.4, –)
				Vitamin A	370–538 mcg (41.1–59.8, 57.6–83.7)
				Vitamin C	13–22 mg (15.3–25.9, 18.4–31.1)
				Calcium	606–869 mg (51–72, 61–87)
				Iron	15.6–16.6 mg (55.6–83.3, 66.7–100)
				Zinc	10–11 mg (83.3–110, 100–132.5)
Madanijah et al. (52), Indonesia (Bogor)	Cross-sectional	Lactating women; between 50 and 180 days after delivery; rural area; 220 subjects	FFQ; foods only	Energy	1,570–1,879 kcal (60.9–72.9, –)
				Protein	50–61 g (62.5 −76.3, –)
				Vitamin A	421–517 mcg (44.3–54.4 : 62–76.2)
				Vitamin C	18–36 mg (15–30, 18–36)
				Calcium	558–803 mg (47–67, 56–80)
				Iron	19.6–21.3 mg (111.1–116.6, 133.3–140)
				Zinc	10–13 mg (76.9–100, 92.3–120)
Mahdy et al. (53), Malaysia (Kuala Lumpur)	Case-control	Post-partum women; pre-eclampsia and normal pregnancy groups; urban area; 75 subjects	3 days dietary recall; foods and supplement	Calcium	446–904 mg (45–90, 53–108)
Marsubrin et al. (54), Indonesia	Longitudinal study	Lactating women; babies were born ≥32 weeks and or birthweight < 1,500 g; 30 subjects	FFQ, single 24-h recall and weekly food records; foods only	Energy	1,679–1,825 kcal (67.7–73.6, –)
				Carbohydrate	240.7–270.6 g (62.5–70.3, –)
				Protein	57.0–64.6 g (71.3–80.8, –)
				Fat	59.4–67.0 g (95.5–107.7, –)
Mohamed et al. (55), Malaysia (Kota Bharu)	Cohort	Pregnant women; 14–24 gestational weeks; urban area; 102 subjects	Single 24-h dietary recall; foods and calcium	Calcium	514–552 mg (49, 58)
Mutalazimah et al. (56), Indonesia (Boyolali)	Cross-sectional	Pregnant women; second and third trimester; rural area; 164 subjects	SQ-FFQ and three days dietary recall; foods only	Energy	1,349 kcal (52.9, –)
				Protein	48 g (68.6, –)
				Carbohydrate	143.9 g (35.9, –)
				Fat	44.4 g (66, –)
Nadimin et al. (57), Indonesia (Makassar)	RCT	Pregnant women; second trimester, rural area; 70 subjects	Single 24-h dietary recall; foods and supplement	Energy	2,077–2,096 kcal (81.4–82.2, –)
				Protein	68–71 g (97.1–101.4, –)
				Vitamin A	1,075–1,315 mcg (119.4–146.1, 167.2–204.6)
				Vitamin D	10–11 mcg (66.7–73.3, 100–110)
				Vitamin E	6–6.4 mg (40–42.7, 48–51.2)
				Thiamine	0.6–0.7 mg (45.7–46.4, 54.9–55.7)
				Riboflavin	0.9 mg (62.9–65.7, 75.4–78.9)
				Pyridoxine	1.2–1.3 mg (64.7–65.7, 77.7–78.9)
				Folate	154.6–173.1 mcg (25.8–28.9, 32.2–36.1)
				Cobalamin	3.2–4.4 mcg (71.8–98, 86.1–117.6)
				Iron	7.1–8.7 mg (26.2–32.2, 31.5–38.6)
				Zinc	6.75–8.69 mg (56.25–72.4, 67.5–86.9)
				Calcium	384–385 mg (32, 38–39)
				Phosphorus	949–1,032 mg (136–147, 164–178)
Nahrisah et al. (58), Indonesia (Aceh)	Cross-sectional	Pregnant women; 14–20 weeks; rural area; 158 subjects	Single 24-h dietary recall; foods only	Copper	0.66 mg (66, 82.5)
				Folate	117.1 mcg (19.5, 24.4)
				Iron	9.84 mg (36.4–65.6, 43,7–78,7)
				Vitamin A	416.4 mcg (46.3, 64.8)
				Vitamin E	4.9 mg (32.7, 39.2)
				Riboflavin	0.7 mg (56.2, 67.4)
				Pyridoxine	0.8 mg (42.1, 50.5)
				Cobalamin	2.1 mcg (46, 55.2)
				Vitamin C	43.1 mg (50.7, 70.8)
				Zinc	5.03 mg (41.91, 50.3)
Nahrisah et al. (59), Indonesia (Aceh)	Quasi experimental	Anemic pregnant women; rural area; 70 subjects	FFQ; foods only	Iron	3.8–6.5 mg (14.07–36.11, 16.8–43.3)
de Pee et al. (60), Indonesia (Bogor)	RCT	Lactating women; rural area; 104 subjects	Single 24-h dietary recall; foods only	Energy	2,436–2,557 kcal (91.9–96.5, –)
				Carbohydrate	376–431 g (90.4–103.9, –)
				Carotenoids	337–351 RE
				Fat	63–73 g (93.8–108.6, –)
				Iron	13 mg (72.2, 86.6)
				Protein	63–65 g (78.8–81.3, –)
				Retinol	16–19 RE
Persson et al. (61), Indonesia (Purworejo)	Cohort	Pregnant women; rural area; 122 subjects (first trimester), 406 subjects (second trimester), 356 subjects (third trimester)	Multiple 24-h dietary recall (3–6 non-consecutive days); foods only	Energy	1,608–1,968 kcal (66.2–77.2, –)
				Protein	39.6–48.9 g (64.9 −69.9, –)
				Fat	38.6–45.4 g (57.4–67.5, –)
				Carbohydrate	282–347 g (73.2–86.8, –)
				Vitamin A	2.3–2.4 mcg (0.3, 0.4)
				Vitamin C	1.6–1.8 mg (1.9–2.1, 2.3–2.5)
				Thiamine	0.7–0.8 mg (47.1–58.6, 56.6–70.3)
				Iron	1.7–1.8 mg (2–2.1, 2.3–2.5)
				Calcium	316–380 mg (26.3–31.6, 31.6–38)
Persson et al. (62), Indonesia (Purworejo)	Cohort	Pregnant women; rural area; 154 subjects (first trimester), 450 subjects (second trimester); 420 subjects (third trimester)	Multiple 24-h dietary recalls (3-6 non-consecutive days); foods and supplement	Vitamin A	363–430 mcg (40.3–47.8, 56.5–66.9)
Rahmannia et al. (63), Indonesia (Sumedang)	Cross-sectional	Lactating women; 2–5 months postpartum; rural area; 121 subjects	12-h daytime weighed food intakes + 12-h maternal recall; foods only	Energy	2,165 kcal (85.7, –)
				Protein	70.7 g (88.4, –)
				Fat	57.8 g (86, –)
				Carbohydrate	338 g (85.2, –)
				Calcium	613 mg (51, 61.3)
				Folate	618 mcg (82.4, 103)
				Iron	17.9 mg (101.6, 122)
				Niacin	12.8 mg (75.3, 97.9)
				Riboflavin	1.7 mg (106.3, 127.5)
				Thiamin	1.4 mg (93.3, 112)
				Vitamin A	501 mcg RE (52.7, 73.8)
				Cobalamin	2.5 mcg (50, 60)
				Pyridoxine	1.3 mg (68.4, 82.1)
				Vitamin C	38 mg (31.7, 38)
				Zinc	12.8 mg (98.4, 118.1)
Savitri et al. (64), Indonesia (Jakarta)	Cohort	Pregnant women; exposed to fasting during Ramadan; urban area; 96 subjects	Single 24-h dietary recall; foods only	Energy	1,527–1,986 kcal (60–77.9, –)
				Protein	52.5–68.7 g (75–98, –)
				Fat	42–69.2 g (622.4–102.8, –)
				Carbohydrate	196.3–281.7 g (49.1–70.4, –)
				Fiber	9–10.4 g (25–28.8, –)
				Vitamin A	1,050–2,442 mg (116.6–271.4, 163.3–379.9)
				Carotene	0 mg (0, 0)
				Vitamin E	0–0.5 mg (0–3.33, 0–4)
				Vitamin C	58.7–78.7 mg (69.1–92.6, 82.9–111.1)
				Thiamine	0.5–0.9 mg (35.7–64.3, 42.9–77.1)
				Riboflavin	0.1–1.2 mg (10–85.7, 12–102.9)
				Pyridoxine	1.2–1.7 mg (63.2–89.5, 75.8–107.4)
				Folate	0–2.8 mcg (0–0.7, 0–0.8)
				Iron	8–14.3 mg (29.6–52.9, 35.5–63.5)
				Zinc	6.3–9.7 mg (52.5–97, 63–116.5)
				Calcium	379–621 mg (32–52, 38–62)
				Phosphorus	647–1,152 mg (92–165, 111–199)
				Sodium	285.9–632.3 mg (19.06–42.1, 19.06–42.1)
				Potassium	1,583–1,978 mg (33.6–42, 33.6–42)
				Magnesium	222.6–282.7 mg (65.4–83.1, 76.7–97.4)
Sulchan et al. (65), Indonesia (Karesidenan Pati, Semarang, Surakarta, Kedu, Banyumas and Pekalongan)	Cross-sectional	Lactating women; children < 36 months old; rural area; 720 subjects	Single 24-h recall; foods only	Vitamin A	319 RE (33.6, 47)
Suprapto et al. (66), Indonesia (Karanganyar)	RCT	Pregnant women; 13–28 weeks; rural area; 84 subjects; foods and supplement	Single 24-h dietary recall; foods and supplement	Energy	1,468–2,045 kcal (57.6–80.2, –)
				Protein	48.1–66 g (68.7–94.3, –)
				Iron	10.1–20.8 mg (37.40–115.5, 44.89–138.6)
				Vitamin A	505.5–15,856 mcg (56.2–176.2, 78.6–246.7)
				Riboflavin	0.5–1.6 mg (38.5–123.1, 46.2–147.7)
				Vitamin C	120.2–263.4 mg (141.1–309.9, 169.7–371.9)
Sutrisna et al. (67), Indonesia	Cross-sectional (secondary data)	Pregnant women; estimate from Riskesdas and SKMI data; national level; 578 subjects	Single 24-h dietary recall; foods only	Iodine	16.2–71.5 μg (7.3–32.5, 10.1–44.6)
Tan et al. (68), Malaysia (Kajang and Seng)	Cross-sectional	Lactating women; infant older than 2 weeks; urban area; 20 subjects	Single 24-h recall; foods only	Calcium	940 mg (94, 113)
				Phosphorus	664 mg (95, 114)
				Potassium	7.2 g (138.5, 153.1)
				Sodium	1,330 mg (88.7, 88.7)
				Magnesium	382 mg (123.2, 131.7)
				Iron	26 mg (123.8, 148.5)
				Zinc	8.5 mg (89.5, 107.3)
				Copper	800 μg (61.5, 80)
				Manganese	1.9 mg (73.1, 73 AI)*
				Selenium	26 μg (76.5, 91.7)
				Iodine	140 μg (70, 98)
				Chromium	32 μg (71.1, 45 AI)*
				Molybdenum	35 μg (70, 36)
Tan et al. (69), Malaysia (Selangor and Kuala Lumpur)	Cross-sectional	Pregnant women; >28 weeks; urban area; 429 subjects	SQ-FFQ; foods only	Energy	1,935 kcal (83.7, –)
				Carbohydrate	375.4 g (73.6, –)
				Protein	80.7 g (103, –)
				Fat	78 g (72.3, –)
				Iron	243 mg (900, 1,080)
				Folate	1,942 mcg (323.7, 420.7)
				Vitamin C	379.6 mg (474.5, 569.4)
				Calcium	1,014 mg (101, 122)
Wibowo et al. (70), Indonesia (Jakarta)	RCT	Pregnant women; 8–12 weeks of gestation; urban area; 46 subjects (intervention), 58 subjects (control)	Single 24-h dietary recall; foods only	Energy	1,339–1,352 kcal (52.5–53, –)
				Protein	47–49.8 g (77–81.6, –)
				Vitamin A	245–819 mcg (27.2–91, 38.1–127.4)
				Vitamin D	0–0.5 mcg (0–3.3, 0–5)
				Zinc	2.55–15.7 mg (21.25–28, 25.5–33.7)
				Iron	19–22.1 mg (35,55–53,33, 42.67–64)
				Folate	225–251 mcg (37.5–41.8, 46.9–42.3)
				Cobalamin	1–1.2 mcg (22.2–26.7, 26.7–32)
				Vitamin C	30.2–42.2 mg (35.5–49.6, 42.6–59.6)
Woon et al. (71), Malaysia (Selangor and Kuala Lumpur)	Cohort	Pregnant women; >28 weeks; urban area; 535 subjects	1-month FFQ; foods only	Vitamin D	10.2 mcg (68, 102)
Yeop et al. (72), Malaysia (Petaling)	Cross-sectional	Pregnant women; first trimester; urban area; 396 subjects	SQ-FFQ; foods only	Calcium	632 mg (63.2, 75.84)
Yong et al. (73), Malaysia (Negeri Sembilan)	Cross-sectional	Pregnant women; 13–26 weeks; 314 subjects	SQ-FFQ; foods and supplement	Energy	1,376 kcal (64.9, –)
				Calcium	800 mg (80, 96)
				Vitamin D	11.5 mcg (76.7, 115)
Yusrawati et al. (74), Indonesia (Padang)	Case-control	Pregnant women; >20 weeks; preeclampsia case group (70 subjects); normotensive control group (70 subjects)	FFQ; foods only	Energy	1,172–1,269 kcal (45.9–49.8, –)
				Protein	42.4–54.9 g (60.6–78.4, –)
				Carbohydrate	163.5–219.5 g (54.9–62.1, –)
				Fat	26.8–32.4 g (39.8–48.1)
				Calcium	936 mg (780, 936)
				Phosphorus	714.6 mg (102, 123)
				Iron	11.1 mg (41.1, 49.3)
				Zinc	5.5 mg (45.8, 55)
				Natrium	533.3 (35.6, 35.3)
				Potassium	732 mg (15,6, 15.6)
				Magnesium	210 mg (63,6, 72.4)
				Vitamin A	544.9–763.9 (60.5–84.9, 85–118.8)
				Folate	108.6–163.5 mcg (18.1–27.3, 22.6–34.1)
				Thiamin	0.7 mg (50, 60)
				Riboflavin	0.9–1.1 mg (67.1–78.6, 80.6–94.3)
				Niacin	4.3–5.8 mg (23.9–32.2, 28.7–38.7)
				Pyridoxine	1.1–1.2 mg (57.9–63.2, 69.5–75.8)
				Cobalamin	1.1–2.8 mcg (24.4–62.2, 29.3–75)
				Vitamin C	43.4–68.2 mg (51.1–80.2, 61–96)
				Vitamin E	1.9–3.6 mg (12.7–24, 15.2–28.8)
Zaleha et al. (75), Malaysia (Selangor)	Cross-sectional (validation study)	Pregnant women; first trimester; urban area; 79 subjects	FFQ and 3 days dietary recall; foods only	Vitamin D	2.9–3.7 mcg (19.6–24.7, 29.4–37)

a ALA, alpha-linolenic acid;

b DHA, docosahexaenoic acid;

^c^EPA, eicosapentaenoic acid;

^*^AI, adequate intake; FFQ, food frequency questionnaire; SQ-FFQ, semi-quantitative food frequency questionnaire.

### Data analysis

Studies of selected nutrients (protein, vitamin A, vitamin D, vitamin C, iron, zinc, and calcium) that provided mean and standard deviation without data transformation (e.g. logarithm transformation) were included in the meta-analysis. Standardized mean differences were calculated with the following criteria; (1) larger sample size was chosen if the study divided the subject into several groups; (2) data conversion to mean and standard deviation was made if the study only provided median, quartile, and interquartile range, according to a study by Wan et al. (76). Meta-analysis was conducted using Review Manager (RevMan) 5.4. In addition, the recommendation of each nutrients were determined by the RDA/RNI and adjusted by requirements of each trimesters for pregnant women; 1st and 2nd 6 months for lactating women. Statistics were presented with mean values and compared with Indonesian RDA and Malaysian RNI levels for macronutrient and micronutrients, labeled with “hypothesized” in forest plots. Heterogeneity among studies was assumed using a random effect model.

### Risk of bias assessment

The risk of bias assessment was modified from Shahar et al. (77) that was specifically developed for nutrition science (i.e dietary assessment). The following information was rated: selection bias (sampling method used and representative of pregnant and lactating women); performance bias (dietary assessment method and measurement of usual intake); and reporting bias (excluded or included over/under-reporters and paid database/primary data collection). Scores ranged from 6 to 9 (low risk of bias), 10 to 13 (moderate risk of bias), and 14 to 16 (high risk of bias). The table was provided in Supplementary Table 2.

## Results

### Summary of research

In total, 2,258 articles were screened. After removing 655 duplicates, 1,603 articles were selected for the title and abstract review. Finally, 57 articles were assessed for full-text review. Four papers were excluded because the full texts were not found. Of 53 articles selected and presented in tables, 36 articles identified pregnant women and 17 mentioned lactating women as the subjects. Among these, 20 studies were carried out in Malaysia and 33 in Indonesia (Table 1). Most studies assessed dietary intakes using a semi-quantitative food frequency questionnaire (SQ-FFQ), while the remaining studies used single 24-h recall, repeated 24-h recall, food records, and FFQ.

### Energy intake

Studies in Indonesia and Malaysia reported various energy intake (kcal/day) profiles among pregnant and lactating women. Assessments of energy intake among pregnant women in Malaysia were mainly conducted in the second and the third trimesters. Daily energy intakes in the second and the third trimesters ranged from 1,376 to 2,310 kcal (64.9–109% RNI), and from 2,021 to 2,042 kcal (87.5–87.9% RNI), respectively. In contrast, energy intake among pregnant women in Indonesia barely achieved 80% RDA. The first trimester of pregnancy had the lowest daily energy intake compared to other trimesters, ranging from 1,166 to 1,608 kcal (45.7–66.2% RDA). The energy intake range of the second trimester of Indonesian pregnant women was 1,269 to 2,096 kcal (49.8–82.2% RDA). Only one out of seven studies found the energy intake of the second trimester to be >80% RDA (57). Likewise, energy intake was also higher in the third trimester compared to the first and the second trimester ranging from 1,500 to 2,443.8 kcal (58.8–95.8% RDA).

Of three studies among Malaysian lactating women, Khor et al. (45) indicated the energy intake of lactating women 2–16 weeks after post-partum was between 94 and 105.6% RNI. In contrast, two other studies (2, 3) showed low energy intake in this population (<80 %RNI). In Indonesia, energy intake among lactating women ranged from 1,482 to 2,556 kcal (55.9–96.5% RDA). Marsubrin et al. (54) demonstrated a decreasing energy intake among lactating women through the weeks with an intake of <80% RDA.

### Protein intake

All four studies conducted among the second and third trimesters of Malaysian pregnant women (4–7) showed a tendency toward high protein consumption (>90% RNI; range 69.9–83.9 g; 90.8–108% RNI). The average protein intake among Indonesian pregnant women ranged widely (42–106 g; 46.7–118% RDA) across the country. The highest protein intake was among pregnant women residing in West Sumatera (106.3 g; 118.1% RDA). The mean intake of protein in the first trimester was significantly lower (40 g; 65.6% RDA) than in the second trimester (47–71 g; 67.1–101.4% RDA) and the third trimester (48.9–106 g; 69.9–118% RDA) (12).

In Malaysia, protein intake among lactating women exceeded 80% of RNI (37.3–100.8 g; 51.8–142% RNI). Only one study in 1985 found a low protein intake in this population (46). Moreover, Khor et al. (45) reported an increase in protein intake according to three-time stages of lactation (83.6–94.3 g), which exceeded the respective RNI. Studies in Indonesia for protein intake in lactating women showed varied results, with most of the studies indicating a protein intake of 70% RDA. Recently, Marsubrin et al. (54) showed a decline in protein intake during the 4-week of lactation (64.6–57 g; 80.8–71.3% RDA).

### Carbohydrate and fiber intake

Carbohydrate intake in Malaysian pregnant women did not meet the recommendation with a range of 245.6 to 306.4 g (63.8–81.9% RNI) (48, 49). Five studies among Indonesian pregnant women exhibited that carbohydrate intakes were > 60% RDA, but they failed to meet 100% RDA. Meanwhile, other studies showed intakes of this nutrient were < 60% RDA (35.9–54.9% RDA) (56, 64, 74, 78). Persson et al. (61) showed that carbohydrate intake changed significantly across the trimesters of pregnancy (282–347 g; 73.2–86.8% RDA).

Overall, the carbohydrate intake of lactating women in Indonesia and Malaysia failed to meet 100% of the recommendation. Malaysia's studies reported a wide range of carbohydrate intake among lactating women (36.8–94.4% of RNI; 139.8–368.2 g) (29, 42, 45, 46). Khor et al. (45) recently declared the carbohydrate intake of this population achieved > 80% RNI. Indonesia's carbohydrate intake of lactating women ranged from 240.7 to 431 g (62.5–103.9% RDA). Only one study among lactating women in Indonesia conducted by de Pee et al. (60) in 1995 showed a carbohydrate intake reaching > 100% RDA. Studies on fiber intake among pregnant women in both countries were limited and the existing evidence consistently showed inadequate intake of fiber. In Indonesia, fiber intakes ranged from 25 to 28.9% RDA. Likewise, fiber intakes among Malaysian pregnant women ranged from 18.3 to 49.3% RNI. However, no studies for fiber intake among lactating women in Indonesia and Malaysia were found.

### Fat intake

Fat intake among pregnant women in Indonesia and Malaysia barely achieved 100% of the recommendation. In Malaysia, the fat intake among pregnant women failed to meet at least 80% RNI with a range of 69–72.4% RNI, while in Indonesia, the range of fat intake was wider, from 26.7 to 162.8% of RDA with two (12, 64) out of 10 studies revealing a fat intake of > 100% RDA (162.8% and 102.8%, respectively). Two studies by Angkasa (27, 28) showed the fat intake among pregnant women in the third trimester living in urban Jakarta was > 90% RDA (94.2–95.4%). Studies describing the total omega-3 fatty acid intake were limited only among Indonesian pregnant women with the mean intake of 1.3 g (96.4% RDA).

Studies in Malaysia indicated that a fat intake among lactating women ranged from 32.7 to 77.1 g (41.4–97.6% RNI). Saturated fat intake data was only found among Malaysian lactating women showing the intakes were consistently below the recommendation (2.9–17.3 g; 11–72.1% RNI). However, the fat intake among lactating women in Indonesia was higher than in Malaysia with a range of 57.8–73 g (86–108.6% RDA) and most intakes exceeded 80% RDA and reached 100% RDA (12, 64). A study by Angkasa et al. reported that Indonesian lactating women living in Jakarta had excessive fat intake of >30% from the total energy intake (27).

### Fat-soluble vitamins

Vitamin A intake among pregnant and lactating women was the most studied fat-soluble vitamin. Ten studies assessing vitamin A intake among Indonesian pregnant women were mainly conducted in rural areas of Java Island with an overall range of 0.3–176.2% RDA (0.4–246.7% EAR). The lowest intake of vitamin A was 2 μg/day and the highest was 505.5–1,585.7 μg/day (61, 66). Some of the studies did not mention the gestational age of the pregnant women. A randomized trial among first-trimester pregnant women revealed that the average intake of vitamin A reached 27.2% RDA (38.1% EAR) for the intervention and 91% RDA (12.74% EAR) for control group (70). Three studies with subjects in the second trimester showed a range of vitamin A intake from 46.3 to 146.1% RDA (64.8–204.1% EAR). Five studies on vitamin A intake among lactating women were only available in Indonesia (rural areas of Java Island) with an intake range of 40.7–54.4% RDA (57–76.2% EAR). Only three studies on vitamin A intake among Malaysian pregnant women took place in health care facilities of urban or suburban areas, with one study in the second-trimester (37), one study in the third-trimester (48), and one study in both the second and the third-trimester of pregnancy (49), but none of the studies reported the intake of lactating women. These studies reported that the vitamin A intake among pregnant women met the minimum recommendation ranging between 119.9 and 148.6% RDA (167.8–208.1% EAR).

Four Indonesian studies (25, 41, 57, 70) and five Malaysian studies (8–12, 22, 31, 47, 71, 75) demonstrated that vitamin D intakes among pregnant women were < 100% RDA/RNI, and mostly could not meet the EAR. The vitamin D intake in Indonesia ranged from 0% to 73.3% RDA (110% EAR), which was similar to the Malaysian intake ranging from 19.6% to 76.7% RDA (29.4–115% EAR). One Indonesian study expressed vitamin D intake in mcg 1,000 kcal/day (5.6 mcg 1,000 kcal/day) (41); therefore, the %RDA and %EAR was not calculated. No data was found for this nutrient among Indonesian and Malaysian lactating women.

Four studies reported vitamin E intake among Indonesian pregnant women to be between 0–42.7% RDA (0–51.2% EAR). These studies were conducted among the second trimester of anemic pregnant women (58, 59), during Ramadan fasting (64), using a case-control design among pregnant women with preeclampsia history and gestational age of >20 weeks (74), and with an experimental design study among the second trimester pregnant women (57). The lowest intake of vitamin E was among urban pregnant women who were exposed to the Ramadan fasting month (64). Data on vitamin E were not available for Indonesian lactating women as well as Malaysian pregnant and lactating women. No studies provided vitamin K intake among pregnant and lactating women in Indonesia and Malaysia.

### Water-soluble vitamins

Vitamin C was the most common nutrient intake assessed among pregnant and lactating women in Indonesia and Malaysia, while studies on niacin, pyridoxine, folate, and cobalamin intakes were lacking. Data on water-soluble vitamin intake including thiamine, riboflavin, niacin, pyridoxine, folate, cobalamin, and vitamin C were scarce among lactating women in Malaysia.

Thiamine intake among Indonesian pregnant women as reported in five studies was predominantly < 60% RDA (43, 57, 64, 74, 75) ranging from 35.7 to 64.3% RDA (42.9–77.1% EAR). The majority of these studies were conducted among second- and third-trimester pregnant women. The highest thiamine intake was observed in a study conducted among pregnant women during Ramadan fasting (64). Three studies from West Java (14, 28, 58) showed the thiamine intake among Indonesian lactating women ranged from 66.7 to 93.3% RDA (80–112% EAR) which was higher than the intake of pregnant women. Compared to Indonesian data, three studies indicated a greater achievement to satisfy the recommendation of thiamine intake among second- and third-trimester Malaysian pregnant women [78.6% RNI (94.3% EAR)−1,443% RNI (1,731% EAR)] (37, 48, 49).

The range of riboflavin intake among second- and third-trimester Indonesian pregnant women in West Java was 38.5–123.1% RDA (46.2–147.7% EAR). Only one study by Suprapto et al. (66) found that the riboflavin intake among pregnant women met the Indonesian RDA and exceeded 100% EAR. Riboflavin intake among Malaysian pregnant women also exceeded the RNI as reported by two studies of Loy et al. (48, 49) and one study by Sawal Hamid et al. (37) with the lowest intake being 114.3% RNI (37). Studies among Indonesian lactating women showed the riboflavin intake ranged between 75 and 106.3% RDA (90–127.5% EAR) (14, 36, 63). No study was found on riboflavin intake among Malaysian lactating women.

Studies on niacin, pyridoxine, folate, and cobalamin intakes were fewer than studies of other vitamins. A case-control study among Indonesian pre-eclampsia and normotensive pregnant women reported that niacin intake achieved only 23.9–32.2% RDA (28.7–38.7% EAR) (74). Niacin intake among Indonesian lactating women achieved higher percentage of RDA compared to the pregnant women. However, this nutrient intake did not meet the recommended EAR, with a highest intake of 75.3% RDA (97.9% EAR). A study in Malaysia demonstrated a deficient niacin intake among pregnant women (6.1% RDA/7.9% EAR) (37), whereas two other studies reported greater percentages of RNI with the highest intake of 88.3% RNI (114.8 EAR) (48, 49). Pyridoxine intake among Indonesian pregnant women ranged from 63.2 to 65% RNI (75.8–78.9% EAR) (57, 58, 64, 74). Meanwhile, the pyridoxine intake among lactating women ranged from 57.9 to 68.4% RNI (69.5–82.1% EAR) (14, 36, 63). Five studies (57, 58, 64, 70, 74) found the folate intake among Indonesian pregnant women did not achieve even 50% RDA. The lowest and the highest folate intake among pregnant women was 0 (64) and 41.8% RDA (52.3% EAR) (70), respectively. A study (58) among Indonesian lactating women reported the folate intake to be 82.4% RNI (103% EAR). In contrast, a study among Malaysian pregnant women reported the folate intake to exceed the recommendation (323.7% RNI/420.7% EAR) (69). Cobalamin intake among Indonesian pregnant women was low as retrieved from four studies (57, 59, 70, 74) with the intake ranging from 22.2 to 98% RDA (26.7–117.6% EAR). Only one study reported that cobalamin intake reached 98% RDA and satisfied the EAR (57). Similarly, cobalamin intake among Indonesian lactating women only met half of the EAR; the highest intake was 50% RDA (60% EAR) (14, 36, 63).

The range of vitamin C intake among Indonesian pregnant women stretched from 1.9 to 309.9% RDA (2.3–371.9% EAR). Four studies in hospitals or health facilities reported the mean vitamin C intake among Malaysian pregnant women. One study in second-trimester pregnant women demonstrated an intake of 156.3% RNI (187.5% EAR) (37), two studies in the third trimester (48, 69) showed an intake range of 151.5–474.5% RNI (181.8–569.4% EAR), and one study in the second to the third trimester indicated intake between 120.5 and 159% RNI (144.6–190.8% EAR) (49). These numbers demonstrated that vitamin C intake in Malaysian pregnant women was noticeably higher than the Indonesians. Two studies among Indonesian lactating women residing in West Java (52, 63) reported the range of vitamin C intake to be from 15 to 31.7% RDA (18–38% EAR). In contrast with Malaysian data, most Indonesian studies showed that the vitamin C intake was mainly below the EAR. No studies reported intake of vitamin C among Malaysian lactating women.

### Macro-minerals (calcium, magnesium, phosphorous, potassium, and sodium)

Among studies reporting the macro-mineral intake, calcium was the most studied in both Indonesian and Malaysian pregnant women (18 studies; nine studies in each country), which were commonly performed in the second and the third trimesters of pregnancy (12, 35, 37–39, 43, 48, 49, 51, 53, 55, 57, 61, 64, 69, 72–74). Indonesian studies showed the range of calcium intake among pregnant women to be from 14% RDA (17% EAR) to 780% RDA (936% EAR) (43, 74). Meanwhile, calcium intake among Malaysian pregnant women ranged from 45% RNI (53% EAR) to 101% RNI (122% EAR) (53, 69). Five studies assessed dietary calcium intake among lactating women in both countries (four studies in Indonesia, and one study in Malaysia). One study conducted among Indonesian exclusive breastfeeding women showed the insufficient calcium intake with the range from 39% RDA (47% EAR) (36) to 51% RDA (61.3% EAR) (14). In contrast, Malaysian data showed the calcium intake of 94% RNI (113% EAR) (68).

Two studies assessed the magnesium intake among Indonesian pregnant women in all trimesters, while no study was found in Malaysia. Magnesium intake among Indonesian pregnant women were below RDA (63.6–83.1% RDA), and not meeting the EAR (72.4–97.4% EAR) (64, 74). A study in Malaysia reported that the magnesium intake of Malaysian lactating women exceeded the recommendation (123.2% RNI; 131.7% EAR) (68).

Phosphorus intake among Indonesian pregnant women ranged from 92 to 165% RDA and exceeded the EAR (123–199% EAR) (64). Likewise, the intake of phosphorous among Malaysian pregnant women ranged from 138 to 172% RDA and exceeded the EAR (167–207% EAR) (49). Furthermore, phosphorous intake data among lactating women was only available in Malaysia, which showed the intake reached 95% RNI (114% EAR) (68).

Potassium intakes among pregnant women were below the EAR for Indonesian (15.6–42%) and Malaysian (33–53.1%) (74). The Indonesian lactating women were insufficient in potassium intake (17.6–21.5% RDA/32–39.2% EAR) (36). Meanwhile one study in Malaysia showed that the potassium intake of lactating women achieved 138.5% RNI (153% EAR) (68). Sodium intake among pregnant women in Indonesia showed a wide range from 19 to 157% EAR (35) while sodium intake data among lactating women was only available in Malaysia, with an intake of 88.7% EAR (68).

### Trace elements (iron, iodine, zinc, chromium, manganese, molybdenum, selenium, copper, boron, and chloride)

Iron intake is the most commonly studied trace element intake in both Indonesia and Malaysia [five studies in Malaysian (37, 39, 48, 49, 69) and 12 studies in Indonesian pregnant women (32, 35, 38, 43, 51, 57, 58, 61, 64, 66, 70, 74); five studies in Indonesian (14, 36, 52, 60, 63) and one study in Malaysian lactating women (68)]. The majority of these studies reported that iron intake among Indonesian pregnant women was insufficient and unable to meet 100% EAR. A study among pregnant women in Malaysia showed that iron intake exceeded the recommendation (900% RNI/1,080% EAR), with the minimum intake being 60.3% RDA (72.4% EAR) (37, 39, 48, 49, 69). Two studies from Indonesia showed inadequate iron intake (65.5–72.2% RDA; 78.6–86.6% EAR) (14, 63), while three studies showed adequate iron intake (101.6–116.6% RDA; 122–140% EAR) among lactating women (36, 52, 60). Iron intake among Malaysian lactating women slightly exceeded the recommendation (123.8% RNI/148.5% EAR) (68).

Iodine intake among pregnant women in Indonesia was insufficient, with the highest intake being 32.5% RDA (44% EAR) (67). In contrast, data on lactating women was only available in a Malaysian study with an intake of 73% EAR (68).

Studies about zinc intake among pregnant women were only found in Indonesia, which were conducted in all trimesters throughout pregnancy. Four of seven studies showed inadequate zinc intake among pregnant women in Indonesia ranging from 21.3 to 72.4% RDA (25.5–86.9% EAR) (57, 58, 70, 74), and two studies showed adequate intake of zinc (83.3–110% RDA/100–132.5% EAR) (35, 51). One particular study assessing zinc intake among Indonesian pregnant women showed sufficient range (52.5–97% RDA/63–116.5% EAR) (64). Zinc intake among Indonesian lactating women ranged from 71.5 to 100% RDA (85.8–120% EAR) (14, 36, 52, 63). Similarly, data from Malaysian lactating women showed a zinc intake of 89.5% RNI (107.3% EAR) (68).

Data for chromium, manganese, molybdenum, selenium, boron, and chloride intakes for pregnant women were unavailable in both countries. However, we found a study that assessed intakes of chromium, manganese, molybdenum, and selenium for Malaysian lactating women, reporting 71.1%, 73.1%, 97.2%, and 91.7% of RNI for these nutrients, respectively (68). A study of copper intake among pregnant women was only found in Indonesia, reporting the intake of 66% RDA (82.5% EAR) (58). Meanwhile, copper intake for lactating women was only reported in a Malaysian study, with the intake of 61.5% RNI (68).

### Meta-analysis and publication bias

#### Protein intake

**Figure 2:**
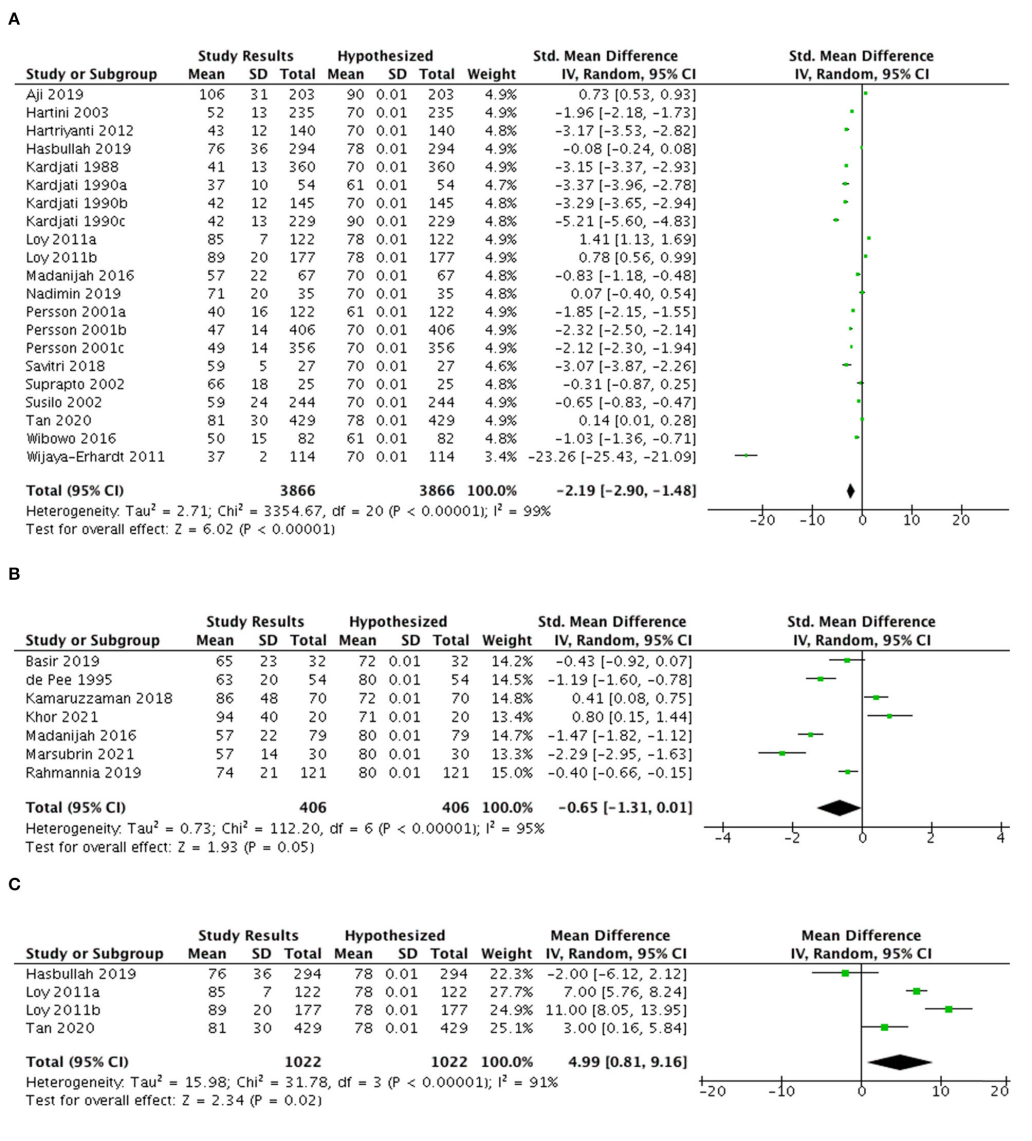
MMeta-analysis of studies on protein intake among **(A)** pregnant women in both countries **(B)** lactating women in Indonesia and Malaysia and **(C)** pregnant women in Malaysia (mean difference in gram).

Figure 2A presents a meta-analysis of 21 studies in Indonesia (17 studies) and Malaysia (four studies), which indicates the overall protein consumption among pregnant women altogether failed to meet the recommendation of this nutrient (SMD: −2.19; 95% CI: −2.90, −1.48). However, a separate analysis of four studies in Malaysia indicated that pregnant women had higher protein consumption as compared to the national intake recommendation for this nutrient (5–7, 13), with an average excess intake of 5 gr/day (106.4% RNI) (Figure 2C). In contrast, the Indonesian studies showed a deficit intake of protein among pregnant women for about 18 g/day.

Seven studies from both countries among lactating women were analyzed (Figure 2B). Overall SMD is −0.65 (95% CI: −1.31, 0.01) suggesting the protein intake of this population tended to be lower than the recommendation. Three studies from Malaysia indicated a higher intake of this nutrient than the national recommendation with the average excess intake of 16.6 gr/day (123.1% RNI). Otherwise, studies in Indonesia reported that lactating women had an average deficit intake of this nutrient for about 11.9 g/day (80.5% RDA).

#### Vitamin A, D, and C intake

**Figure 3:**
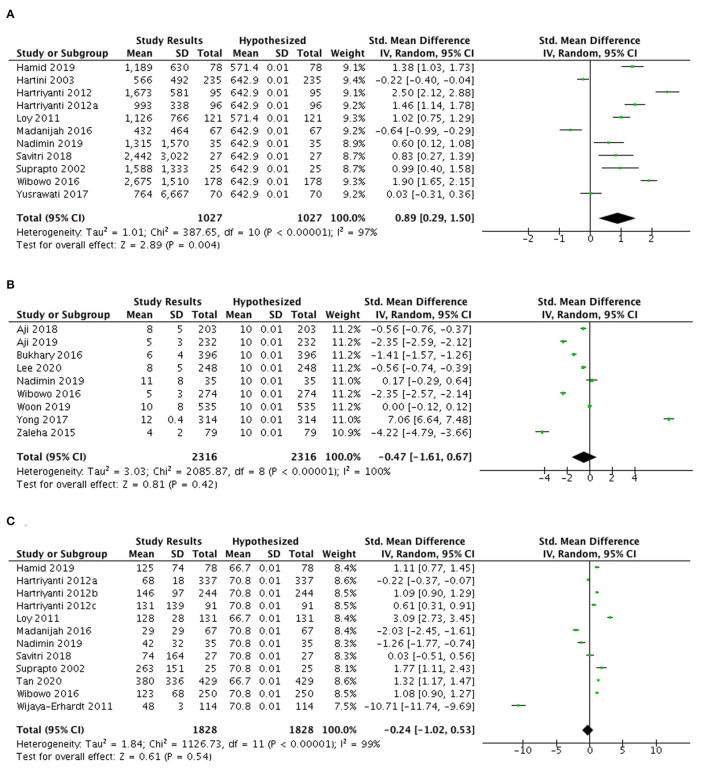
**(A)** Meta-analysis of studies on vitamin A, **(B)** vitamin D, and **(C)** vitamin C intakes among pregnant women in Indonesia and Malaysia.

As shown in Figure 3A, eleven studies were selected for a meta-analysis of vitamin A intake among pregnant women. The vitamin A intake in Indonesia and Malaysia of this population met the EAR levels (SMD 0.89; 95% CI: 0.29, 1.50) with an average excess intake of 428.2 mcg/day.

Nine studies assessing vitamin D intake (mcg/day) among pregnant women in Indonesia and Malaysia were included in the meta-analysis (Figure 3B). The vitamin D intakes in this population in both countries tended not to achieve the recommendation (SMD −0.47; 95% CI: −1.61, 0.67), with an average deficit of 2.36 mcg/day (77.7% EAR). Meanwhile Yong et al. (73) presented a significant 2 mcg/day higher intake of vitamin D among pregnant women as compared to the 100% EAR for this nutrient.

Figure 3C highlights that the overall vitamin C intake among pregnant women in Malaysia and Indonesia was lower than the 100% EAR (SMD −0.24; 95% CI: −1.02, 0.53). The insufficient intake of this nutrient mostly came from Indonesian studies, with an average deficit of 68.73 mg/day (EAR). Nonetheless, three studies from Malaysia reported that the vitamin C intakes were greater than the 100% EAR, with an average intake of 132.9 mg/day (121.4% EAR).

#### Iron, zinc, and calcium intake

**Figure 4:**
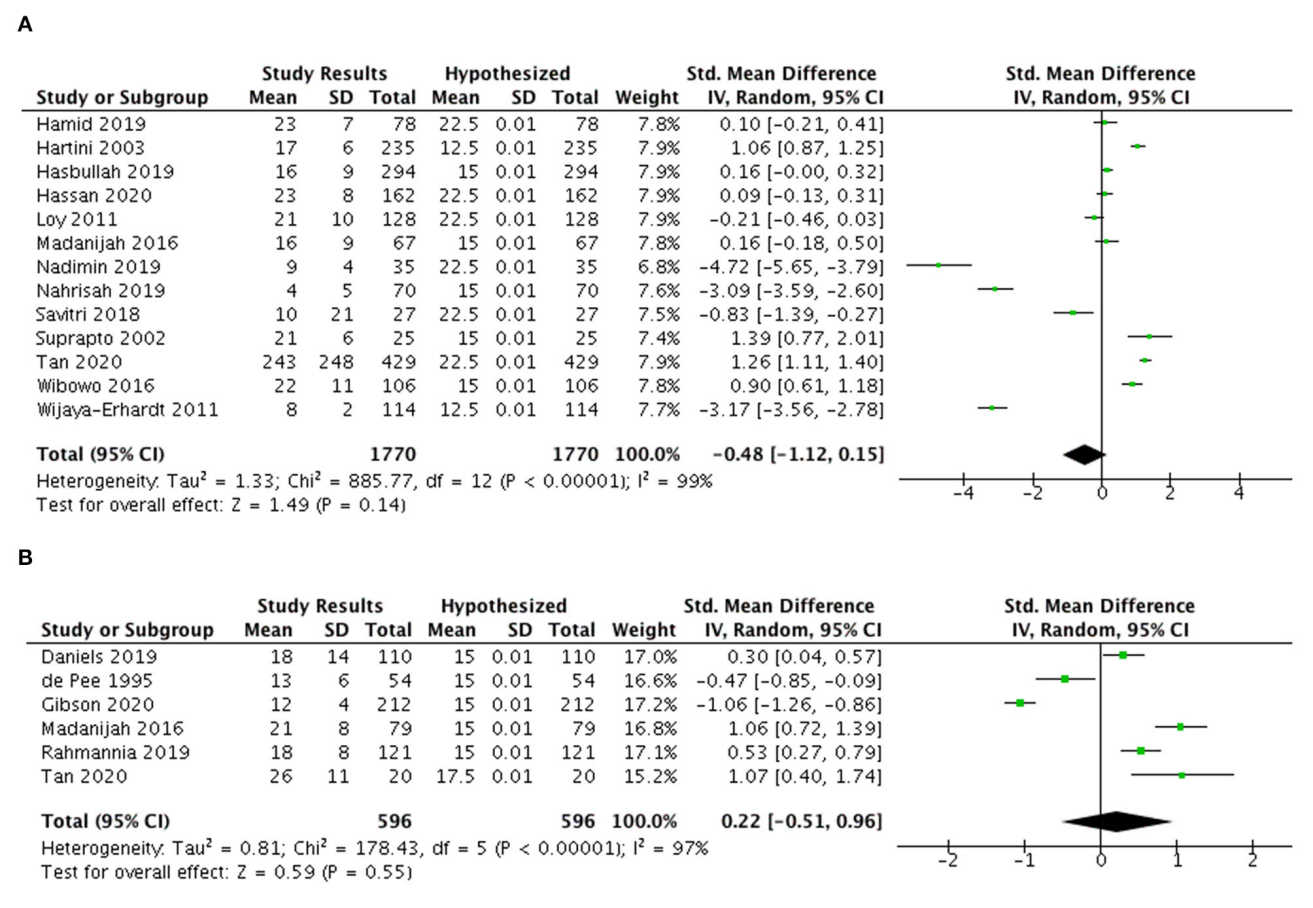
Meta-analysis of studies on iron intake **(A)** among pregnant women and **(B)** lactating women in Indonesia and Malaysia.

A meta-analysis of 13 studies (Figure 4A) showed inadequate iron intake among Indonesian and Malaysian pregnant women with an overall SMD of −0.48 (95% CI: −1.12, 0.15).

On the contrary, six studies among Indonesian and Malaysian lactating women indicated a tendency for higher iron intake than 100% EAR (Figure 4B) with an SMD of 0.22 (95%CI: −0.51, 0.96) and the average excess was 2.32 mg/day in both countries.

**Figure 5:**
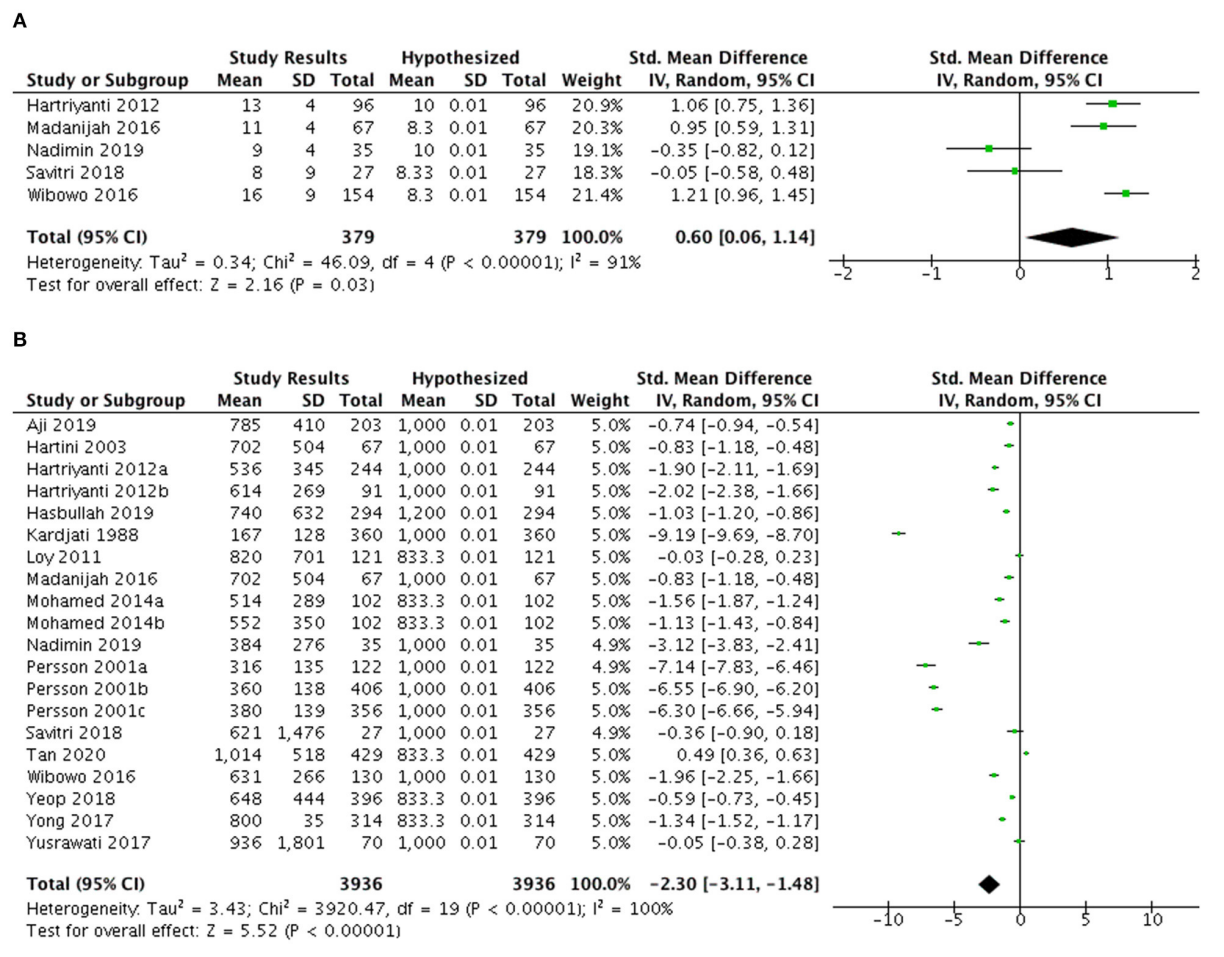
Meta-analysis of studies on **(A)** zinc intake and **(B)** calcium intake among pregnant women in Indonesia and Malaysia.

Figure 5A presents the zinc intakes of Indonesian pregnant women (*n* = 5 studies) that exceeded the optimal EAR levels with average excess of 2.55 mg/day (SMD 0.6; 95% CI: 0.06, 1.14).

Figure 5B highlights the findings of 20 studies from Indonesia and Malaysia indicating the calcium intake among pregnant women was below 100% EAR levels (SMD −2.30; 95% CI: −3.11, −1.48), with an average deficit of 351.83 mg/day.

#### Result of bias

A low to high risk of bias was found in this study. The total score of the bias assessment are shown in Supplementary Table 2. Moreover, funnel plots for certain nutrients included in the meta-analysis are presented in Supplementary Figures 1–3, with distributions of points to the left and right.

## Discussion

The present review discovered that the energy and macro-nutrient intakes among Indonesian and Malaysian pregnant and lactating women (53 studies) were below RDA/RNI. Water- and fat-soluble vitamin intakes failed to meet the recommendation and were mainly lower than 80% EAR, except for vitamin C and A in Malaysian pregnant women. Macro-minerals (calcium and potassium among pregnant women and Indonesian lactating women; magnesium in Indonesian pregnant women) and trace element (iron in pregnant women in both countries; iodine in Indonesian pregnant women and Malaysian lactating women) intakes tended to be insufficient. Moreover, zinc intake was adequate among pregnant women in Indonesia, but no zinc intake study was found in Malaysia. Dietary intakes of these nutrients exceeded 100% EAR (Phosphorus intake for pregnant women in both countries, sodium intake in Malaysian pregnant women, as well as calcium and potassium intakes in Malaysian lactating women). To the best of our knowledge, this study is the first systematic review and meta-analysis of nutrient intake among pregnant and lactating women in Indonesia and Malaysia. A thorough review with a double-screening approach was performed to assure the quality of the data in the systematic review.

### Macronutrient intake

The energy intake of Indonesian and Malaysian pregnant women exhibited a similar pattern of not meeting the national recommendation. A comparable finding was also observed in a study conducted among pregnant women in Thailand (79). On the contrary, Nguyen et al. (80) found that energy intake achieved the RNI among Vietnamese pregnant women with light physical activity but failed to meet RNI for those with moderate physical activity. The inadequate energy intake might be caused by seasonal food availability, cultural prohibition, and lack of knowledge (79–81). Moreover, food restriction during pregnancy could cause mothers to have lower energy intake and lower consumption of diverse foods than the recommendation (82). A study by Ding et al. found that energy and protein intakes among pregnant women decreased along with decreasing economic quintiles (83), which was also supported by a study done in Nepal (84) showing wealthier women were more likely to consume diverse diets than the poorer. This review found that energy intake was lower in the first trimester of pregnancy compared to the second and the third, predominantly due to nausea and vomiting (14, 15, 85).

Despite the need to increase energy requirements among lactating women to produce sufficient breast milk (86), our present review demonstrated that the energy intake among Indonesian and Malaysian lactating women failed to meet the national recommendation. This finding is similar to the study in China indicating almost 83% of lactating women had lower energy intake (83). A study conducted among Indonesian lactating women revealed that low energy intake was caused by the lack of knowledge, being exhausted, and limited time to prepare the foods (87). A socioeconomic factor might also contribute to the inadequate energy intake of lactating women in Indonesia (16). Although the energy intake of lactating women in both socioeconomic conditions was insufficient, much lower energy adequacy was found in low socioeconomic than in high socioeconomic women (17). A few studies in Malaysia indicated that low energy intake among lactating women was affected by food restriction due to a belief that lactating mothers were in a “cold state” after delivery. Restriction of consuming certain types of foods was believed to balance the hot and cold states of the body (29, 88).

Our review and meta-analysis also found that the protein intakes among Malaysian pregnant and lactating women were adequate. Yong et al. reported that Malaysian lactating women had perceived belief that protein rich foods such as meat, cheese, and milk products benefit the infant growth and development (22). The existence of food taboos among pregnant women in some cultures to certain protein source foods such as seafoods may hinder the consumption of these foods. It was believed that these foods contained high cholesterol and caused complications during delivery (29, 88). Furthermore, a high protein intake in pregnant and lactating women in Malaysia followed the same intake pattern of Malaysian adults. A recent review by Shahar et al. (77) indicated that the total protein intake among Malaysian adults remained higher than the national recommendation. The consumption of excessive-high protein food source might develop the risk of diet-related diseases and impact fetal development. However, lower protein intake during pregnancy was associated with adverse effects on the weight and length of the baby at birth (89). Goh et al. stated that Malaysia was under the “substitution phase” of nutrition transition, characterized by shifting in many types of foods without having any changes in the overall energy supply. This condition caused an increase in animal source foods, along with a reduction in legumes, fruits, and vegetables consumptions (17).

This present review discovered that studies of omega-3 and omega-6 fatty acids intakes among pregnant and lactating women in both countries were limited. One study in Indonesian pregnant women indicated inadequate intake of these nutrients. This result was not aligned with a study conducted in China showing the omega-3 fatty acid intake among pregnant women was higher than the recommendation (89). Many studies demonstrated the healthful benefits of omega-3 fatty acids, in increasing gestation length, birth weight and length. Omega-3 fatty acid have anti-oxidative and anti-inflammatory properties that could decrease the risk of preeclampsia (90, 91).

Most studies reported low carbohydrate intakes among pregnant and lactating women in Indonesia and Malaysia (only met 60–80% of RDA/RNI). Lower carbohydrate intake among obese pregnant women had been associated with lower fat mass in the offspring (92). In the GUSTO Study, lower carbohydrate intake among pregnant women in Singapore was associated with lower abdominal, internal tissue in newborn babies (92). Moreover, fiber intakes of Malaysian and Indonesian pregnant and lactating women were low, with a range of 20–40% of RDA/RNI.

### Vitamin intake

We discovered the vitamin intakes among pregnant and lactating women were different between Indonesia and Malaysia. Most studies suggested adequate intakes of vitamin A based on the EAR, except for Indonesian lactating women. Study in Indonesia suggested that although vitamin A intake of the women was adequate (predominantly from vegetables consumption), the serum retinol in this population was low (93). Rahmannia et al. (63) previously highlighted that lactating mothers commonly consumed starchy staples, which provided little contribution to fulfill intakes of vitamins, including vitamin A. Therefore, lactating women in Indonesia are recommended to consume a high dosage of vitamin A supplementation as one of the national nutrition programs. Some studies in several areas in Indonesia reported that most mothers consumed vitamin A supplements (94, 95) but the program coverage of this supplementation was greater in urban areas than in rural areas (96). Lactating women did not consume any vitamin A supplements due to lack of knowledge and information given by the health workers or midwives (94).

Almost all vitamin B intakes were inadequate and could not meet the expected EAR among pregnant and lactating women, particularly in Indonesia. While in Malaysia, our findings suggested that vitamin B intake exceeded the recommendation. Some studies recorded inadequate vitamin B intakes among adults (97) and pre-conception women (98) in Indonesia. Unfortunately, the retrieved studies did not further explain information on vitamin B source foods contributing to the intake. Indonesian typical diet comprises mainly carbohydrate source foods, a small portion of protein source foods, and vegetables rich in vitamin B. Meanwhile, Yong et al. found that the dietary pattern in Malaysia in the early stage of pregnancy was considered prudent, with high intakes of nuts, seeds, legumes, vegetables, and dairy products (13, 99) that may contribute to vitamin B intake.

The mean vitamin C intake among Malaysian pregnant women exceeded the RNI and was greater than the intake of Indonesian pregnant women who had lower intake of this nutrient than the expected vitamin C EAR. A review done by Caut et al. reported that pregnant women in Malaysia frequently adhered to the recommendations for fruits consumption as a source of vitamin C (100). However, another study mentioned that fruit and vegetable consumptions were inadequate in Malaysian pregnant women (49, 101). Previous studies and national reports in Indonesia often mentioned that fruit and vegetable consumption is a major concern in Indonesia. However, studies from Indonesia did not provide further detail on the diet or vitamin C source foods (102).

Vitamin D intake was inadequate among Malaysian and Indonesian pregnant and lactating women. Woon (71) stated that the majority of pregnant women relied on foods as the main source of vitamin D. Fish and fish products, milk and milk products, and eggs were the main food contributing to vitamin D intake among Malaysian pregnant women. Although dietary sources of vitamin D were not commonly and adequately consumed and vitamin D-fortified foods were limited in low-middle income countries, abundant sunlight in Indonesia and Malaysia should allow direct sun exposure to the skin and become a potential source of the vitamin D to meet its requirement. However, vitamin D insufficiency and deficiency were widespread, not only among pregnant women (9, 20, 21), but also across population from newborns (103), children (104), adolescents (105, 106), adults (22), and elderly (107) in these countries. Countries in Asia, the Middle East, and Africa were frequently reported to have a high prevalence of vitamin D deficiencies. Sun-protective behaviors related to culture or religion, such as covering skin, wearing hats, applying sunscreen, and limiting outdoor activities, were the main factors associated with vitamin D deficiencies (108). In contrast, the consumption of vitamin D-rich foods such as oil rich fish and dairy products in European countries was remarkable, although the sunlight exposure was not as much in this region. Existing policies on vitamin D supplementation and food fortification in Europe and other countries such as USA, Canada, and India may facilitate the population to adequately consume this nutrient. This policy was also accompanied with the encouragement to increase outdoor activities which can provide sun exposure to enhance vitamin D synthesis on the skin (109, 110).

Dietary supplements consumption was not a common practice among pregnant and lactating women in Indonesia and Malaysia (9, 19, 20), unless it was given by the health workers. A recent large survey among Malaysian adults found only 28.1% of women were more likely to consume the vitamin/mineral supplements (101). In contrast, 77% of pregnant and 70.3% lactating women in US consumed one or more dietary supplements, which were significantly higher than the consumption of non-pregnant and non-lactating women (111). Study done by Daud et al. reported that Malaysian pregnant women had a good knowledge and awareness on the role of dietary supplements during pregnancy, but only half of the pregnant women took dietary supplements mainly to overcome anemia (112). It was also aligned with the national program in providing iron pills (hematinic) to pregnant women in Malaysia. In Indonesia, the national program of iron-folic acid (IFA) tablets for pregnant women and vitamin A supplementation for lactating women (96) have been established, yet wide-scale supplementation program and study on the application of vitamin D among pregnant women was scarce. Altogether, vitamin D supplementation during pregnancy has not been included in the national program for Indonesia and Malaysia (25, 26, 41, 47).

### Macro-minerals and trace elements intake

The data from Indonesia Health Survey showed an increased prevalence of anemia among pregnant women from 37.1% in 2013 to 48.9% in 2018 (113, 114). Meanwhile, data from National Health and Morbidity Survey in Malaysia showed a declined prevalence of anemia from 2015 to 2019 from 24.6% to 21.3% (115, 116). Most studies showed that the iron intake of pregnant women was below the recommendation of the National Dietary Guidelines in Indonesia and Malaysia. This inadequate intake of iron may cause iron deficiency, which is one of the most prominent causes of anemia among pregnant women. RDA for iron intake during pregnancy is 27 mg per day based on IOM 2001, which is set at a level for women begin storing iron in their early pregnancy. However, a study by Machmud et al. found that pregnant women in Indonesia commonly consumed plant-based protein food, that were considerably low in iron content (117). A study by Sawal Hamid et al. (37) showed that iron and vitamin B3 among pregnant women in Malaysia was difficult to meet the recommendation without supplements or fortified foods. Therefore, supporting the guideline for iron pills supplementation help to increase consumption of iron during pregnancy. In this review, we found that the iron intake was significantly higher than the recommendation in lactating women. Compared to pregnant women, lactating women often consumed more foods as they had better appetite and willingness to produce adequate breastmilk (86).

Our meta-analysis from five studies available only in Indonesia, not in Malaysia, showed that the zinc intake among pregnant women was above the recommendation, in contrast with their iron intake profile. This finding is not in line with the previous study by Lim et al., in which inadequacy of iron intake was often associated with low zinc intake as both nutrients were rich in similar animal protein food sources (118).

Our present study found inadequate calcium intake among pregnant women, which was below the recommended intake based on the National Dietary Guidelines of Indonesia and Malaysia. Similarly, a study on the global trend of micronutrient intake by Beal et al. (119) showed that both iron and calcium were the most insufficient micronutrient intakes. The result from this study was similar to the calcium intake among pregnant women in another South-east Asia country, Thailand, that showed the intake of 602.4 mg/day (120). In contrast, a systematic review and meta analysis by Cormick et al. (121) indicated that the calcium intake in high-income countries ranged from 283 to 2,228 mg/day, which was much higher than the intake in low middle-income countries (210 to 1,631 mg/day).

Assessments of iodine intake were only available in one study among Indonesian pregnant women and one study among Malaysian lactating women, which demonstrated inadequate intake of this nutrient. Iodine intake in Indonesian study was estimated based on the result from the National Health Survey and National Total Diet Study covering urban and rural women (67), whereas in Malaysia iodine intake was evaluated based on the study conducted in certain urban city of Kuala Lumpur (68). In terms of iodine status, Lim et al. (122) discovered that 60% of pregnant women who lived in rural areas of Sabah, Malaysia had median urinary iodine concentrations of <150 μg/L. Meanwhile, a study in suburb area of Central Java, Indonesia, showed that 13.8% of pregnant women had low median urinary iodine concentration (<150 μg/L), this proportion increased in each trimester. The biggest source of daily iodine consumption was iodized salt, while food sources of iodine were less consumed in pregnant women (123). The previous study confirmed that iodized salt and daily supplementation of iodine capsules had similar effects in determining the iodine status and thyroid hormones. This finding underlined the importance of a universal iodized salt program (124).

### Limitations of the study

Few limitations were addressed in this study. As mentioned above, the macro and micronutrient dietary intake data were limited among pregnant and lactating women in Indonesia and Malaysia. Therefore, this review summarized all eligible studies including those with moderate to high risk of bias. Since the recommended intake of pregnant women was different in each trimester, it was challenging to draw the average percentage of excessive or deficit intake in terms of RNI/RDA/EAR in meta-analysis. Thus, we only provided the percentage of certain nutrients which had a similar recommendation. The various nutritional assessment methods applied in the available studies may cause discrepancy in estimating and providing significant range of the dietary intake.

Our result also demonstrated that studies on pregnant women in the first trimester and lactating women were lacking. Thus, future research should consider focusing on these populations, along with more exploration to nutrient of interest with limited data, such as vitamin E, vitamin K, vitamin B, macro-minerals, and trace elements.

## Conclusion

In summary, the overall findings of this comprehensive review of dietary intake indicated that Indonesian and Malaysian pregnant and lactating women had insufficient intake of essential nutrients. Dietary intakes of energy, macronutrients, and micronutrients (vitamin D, vitamin E, water-soluble vitamins, calcium, and iron) of pregnant and lactating women in Indonesia and Malaysia were below the recommendations. Important heterogeneities were observed even between these two countries for specific essential nutrient intakes. Innovative research and targeted programs to address specific deficiencies should be prioritized. Promotive and preventive national programs to improve maternal nutrient intake can be implemented such as promoting maternal diet rich in nutrients even before conception, reevaluating the required supplementation during pregnancy and lactation for specific nutrient deficiencies, enhancing compliance of the current dietary supplementation and initiating the implementation of multi-micro-nutrient supplementation for pregnant women. Fortified and functional food formulations targeted these populations could be explored as the alternative mode in ensuring the nutrient content of specific food products. The utilization of digital technology to educate proper nutrition and promote healthy diet practice for pregnant and lactating women should be enhanced and financed.

## Data availability statement

The original contributions presented in the study are included in the article/Supplementary material, further inquiries can be directed to the corresponding author.

## Author contributions

Conceptualization: RA, DR, VS, and WL. Methodology and validation: RA, DR, and WL. Software, visualization, and project administration: DR. Formal analysis: WL, FB, and RM. Writing—original draft preparation: RA, WL, FB, and RM. Writing—review and editing, supervision, and funding acquisition: RA. All authors have read and agreed to the published version of the manuscript.
