# Supplementary material for: Nutrient intakes of pregnant and lactating women in Indonesia and Malaysia: Systematic review and meta-analysis
Source: Front Nutr. 2023 Mar 30;10:1030343. doi: 10.3389/fnut.2023.1030343 (PMC10098007; doi:10.3389/fnut.2023.1030343)
Supplement: Supplementary file 1 [file Table_1.docx]

**Supplementary Table 1 Database, search strategy, and number of studies**

| Database | Search Strategy | No. of studies |
| --- | --- | --- |
| Pubmed | (indonesia*[Title/Abstract] OR malaysia*[Title/Abstract] OR asia*[Title/Abstract] OR “southeast asia”[Title/Abstract] OR “South East Asia"[Title/Abstract]) AND (pregnan*[Title/Abstract] OR lactati*[Title/Abstract] OR breastfe*[Title/Abstract]) AND (macronutrient*[Title/Abstract] OR micronutrient*[Title/Abstract] OR vitamin*[Title/Abstract] OR carbohydrate*[Title/Abstract] OR protein[Title/Abstract] OR fat*[Title/Abstract] OR fiber[Title/Abstract] OR fibre[Title/Abstract] OR "amino acid"[Title/Abstract] OR omega[Title/Abstract] OR "omega-3"[Title/Abstract] OR "omega-6"[Title/Abstract] OR "linolenic acid"[Title/Abstract] OR "linoleic acid"[Title/Abstract] OR "docosahexaenoic acid"[Title/Abstract] OR "eicosapentaenoic acid"[Title/Abstract] OR "saturated fat"[Title/Abstract] OR "trans fat"[Title/Abstract] OR calorie OR "vitamin A"[Title/Abstract] OR betacaroten* OR carotenoid* OR "vitamin D"[Title/Abstract] OR "vitamin E"[Title/Abstract] OR "vitamin K"[Title/Abstract] OR menaquinone* OR thiamin* OR riboflavin OR niacin OR "pantothenic acid"[Title/Abstract] OR pyridoxin[Title/Abstract] OR "B6"[Title/Abstract] OR biotin OR "folic acid"[Title/Abstract] OR folate[Title/Abstract] OR cobalamin[Title/Abstract] OR B12[Title/Abstract] OR "vitamin C"[Title/Abstract] OR "ascorbic acid"[Title/Abstract] OR "mineral"[Title/Abstract] OR iron[Title/Abstract] OR zinc[Title/Abstract] OR iodine[Title/Abstract] OR calcium[Title/Abstract] OR selenium[Title/Abstract] OR magnesium[Title/Abstract] OR phosporus[Title/Abstract] OR sodium[Title/Abstract] OR potassium[Title/Abstract] OR boron[Title/Abstract] OR manganese[Title/Abstract] OR molybdenum[Title/Abstract] OR chromium[Title/Abstract] OR copper[Title/Abstract]) AND (“nutritional assessment”[Title/Abstract] OR “nutritional deficiency”[Title/Abstract] OR “nutritional status”[Title/Abstract] OR “maternal nutrition”[Title/Abstract] OR intake[Title/Abstract] OR consumption[Title/Abstract] OR “food frequency questionnaire”[Title/Abstract] OR supplementation[Title/Abstract]) | 542 |
| Proquest | AB,TI(((Indonesia* OR Malaysia* OR Asia* OR “Southeast Asia” OR “South-East Asia”) AND (pregnan* OR lactati* OR breastfe*) AND (macronutrient* OR micronutrient* OR vitamin* OR carbohydrate* OR protein OR fat* OR fiber OR fibre OR "amino acid" OR omega OR "omega-3" OR "omega-6" OR "linolenic acid" OR "linoleic acid" OR "docosahexaenoic acid" OR "eicosapentaenoic acid” OR "saturated fat" OR "trans fat" OR “trans-fat” OR calorie OR "vitamin A" OR betacaroten* OR carotenoid* OR "vitamin D" OR "vitamin E" OR "vitamin K" OR menaquinone* OR thiamin* OR riboflavin OR niacin OR "pantothenic acid" OR pyridoxin OR B6 OR biotin OR "folic acid" OR folate OR cobalamin OR B12 OR "vitamin C" OR "ascorbic acid" OR "mineral" OR iron OR zinc OR iodine OR calcium OR selenium OR magnesium OR phosphorus OR sodium OR potassium OR boron OR manganese OR molybdenum OR chromium OR copper) AND (“nutritional assessment” OR “nutritional deficiency” OR “nutritional status” OR “maternal nutrition” OR intake OR consumption OR “food frequency questionnaire” OR supplementation))) | 229 |
| Cochrane review | (Indonesia* OR Malaysia* OR Asia* OR Southeast Asia OR South-East Asia) AND (pregnan* OR lactati* OR breastfe*) AND (macronutrient* OR micronutrient* OR vitamin* OR carbohydrate* OR protein OR fat* OR fiber OR fibre OR "amino acid" OR omega OR omega-3 OR omega-6 OR linolenic acid OR linoleic acid OR docosahexaenoic acid OR eicosapentaenoic acid OR saturated fat OR trans fat OR trans-fat OR calorie OR vitamin A OR betacaroten* OR carotenoid* OR vitamin D OR vitamin E OR vitamin K OR menaquinone* OR thiamin* OR riboflavin OR niacin OR pantothenic acid OR pyridoxin OR B6 OR biotin OR folic acid OR folate OR cobalamin OR B12 OR vitamin C OR ascorbic acid OR mineral OR iron OR zinc OR iodine OR calcium OR selenium OR magnesium OR phosphorus OR sodium OR potassium OR boron OR manganese OR molybdenum OR chromium OR copper) AND (nutritional assessment OR nutritional deficiency OR nutritional status OR maternal nutrition OR intake OR consumption OR food frequency questionnaire OR supplementation) | 11 |
| Cochrane Trial | (Indonesia* OR Malaysia* OR Asia* OR Southeast Asia OR South-East Asia) AND (pregnan* OR lactati* OR breastfe*) AND (macronutrient* OR micronutrient* OR vitamin* OR carbohydrate* OR protein OR fat* OR fiber OR fibre OR "amino acid" OR omega OR omega-3 OR omega-6 OR linolenic acid OR linoleic acid OR docosahexaenoic acid OR eicosapentaenoic acid OR saturated fat OR trans fat OR trans-fat OR calorie OR vitamin A OR betacaroten* OR carotenoid* OR vitamin D OR vitamin E OR vitamin K OR menaquinone* OR thiamin* OR riboflavin OR niacin OR pantothenic acid OR pyridoxin OR B6 OR biotin OR folic acid OR folate OR cobalamin OR B12 OR vitamin C OR ascorbic acid OR mineral OR iron OR zinc OR iodine OR calcium OR selenium OR magnesium OR phosphorus OR sodium OR potassium OR boron OR manganese OR molybdenum OR chromium OR copper) AND (nutritional assessment OR nutritional deficiency OR nutritional status OR maternal nutrition OR intake OR consumption OR food frequency questionnaire OR supplementation) | 196 |
| Scopus | TITLE-ABS-KEY ( ( ( indonesia* OR malaysia* OR asia* OR "Southeast Asia" OR "South-East Asia" ) AND ( pregnan* OR lactati* OR breastfe* ) AND ( macronutrient* OR micronutrient* OR vitamin* OR carbohydrate* OR protein OR fat* OR fiber OR fibre OR "amino acid" OR omega OR "omega-3" OR "omega-6" OR "linolenic acid" OR "linoleic acid" OR "docosahexaenoic acid" OR "eicosapentaenoic acid" OR "saturated fat" OR "trans fat" OR "trans-fat" OR calorie OR "vitamin A" OR betacaroten* OR carotenoid* OR "vitamin D" OR "vitamin E" OR "vitamin K" OR menaquinone* OR thiamin* OR riboflavin OR niacin OR "pantothenic acid" OR pyridoxin OR b6 OR biotin OR "folic acid" OR folate OR cobalamin OR b12 OR "vitamin C" OR "ascorbic acid" OR "mineral" OR iron OR zinc OR iodine OR calcium OR selenium OR magnesium OR phosphorus OR sodium OR potassium OR boron OR manganese OR molybdenum OR chromium OR copper ) AND ( "nutritional assessment" OR "nutritional deficiency" OR "nutritional status" OR "maternal nutrition" OR intake OR consumption OR "food frequency questionnaire" OR supplementation ) ) ) | 1280 |
| Total |  | 2258 |
