# Supplementary material for: Nutrient intakes of pregnant and lactating women in Indonesia and Malaysia: Systematic review and meta-analysis
Source: Front Nutr. 2023 Mar 30;10:1030343. doi: 10.3389/fnut.2023.1030343 (PMC10098007; doi:10.3389/fnut.2023.1030343)
Supplement: Supplementary file 2 [file Table_2.docx]

**Supplementary Table 2 Risk of bias assessment**

| Study | Title of the study | Selection bias |  | Performance bias |  | Reporting bias |  | Total score | Classification of risk of bias |
| --- | --- | --- | --- | --- | --- | --- | --- | --- | --- |
|  |  | Sampling method | Representative of age | Dietary assessment method used | Usual intake measured | considering under/over reported | Primary data collection vs paid database |  |  |
| Aji, 2018, Indonesia (West Sumatera) | The association between lifestyle and maternal vitamin D during pregnancy in West Sumatra, Indonesia | 2 | 1 | 3 | 1 | 2 | 2 | 11 | moderate risk |
| Aji, 2019, Indonesia (West Sumatera) | Vitamin D deficiency status and its related risk factors during early pregnancy: a cross-sectional study of pregnant Minangkabau women, Indonesia | 2 | 1 | 3 | 1 | 2 | 2 | 11 | moderate risk |
| Aji, 2019, Indonesia (West Sumatera) | Low Maternal Vitamin D and Calcium Food Intake during Pregnancy Associated with Place of Residence: A Cross-Sectional Study in West Sumatran Women, Indonesia | 2 | 1 | 3 | 1 | 2 | 2 | 11 | moderate risk |
| Angkasa, 2017, Indonesia (Jakarta) | Inadequate dietary α-linolenic acid intake among Indonesian pregnant women is associated with lower newborn weights in urban Jakarta | 2 | 1 | 3 | 1 | 2 | 2 | 11 | moderate risk |
| Angkasa, 2019, Indonesia (Jakarta) | Validation of a semi-quantitative food frequency questionnaire for estimating dietary omega-3 fatty acids intake among urban Indonesian pregnant women | 1 | 1 | 3 | 1 | 1 | 2 | 9 | low risk |
| Basir, 2019, Malaysia | Dietary intake of mothers who practised traditional confinement during exclusive breastfeeding period; Maternal diet and its association with human milk energy and macronutrient composition among exclusively breastfeeding Malaysian Malay mothers | 1 | 1 | 2 | 1 | 2 | 2 | 9 | low risk |
| Bukhary, 2016, Malaysia (Petaling) | Risk factors for antenatal hypovitaminosis D in an urban district in Malaysia | 2 | 1 | 3 | 1 | 2 | 2 | 11 | moderate risk |
| Daniels, 2019, Indonesia (Sumedang) | Micronutrient intakes of lactating mothers and their association with breast milk concentrations and micronutrient adequacy of exclusively breastfed Indonesian infants | 2 | 1 | 1 | 1 | 2 | 2 | 9 | low risk |
| Erhardt, 2011, Indonesia (Demak) | Fermented soyabean and vitamin C-rich fruit: a possibility to circumvent the further decrease of iron status among iron-deficient pregnant women in Indonesia | 1 | 1 | 2 | 1 | 2 | 2 | 9 | low risk |
| Fikawati, 2014, Indonesia | Comparison of lactational performance of vegetarian and non-vegetarian mothers in Indonesia | 2 | 1 | 3 | 1 | 2 | 2 | 11 | moderate risk |
| Fikawati, 2017, Indonesia | Maternal calorie intake is a significant factor associated with 6 months of exclusive breastfeeding among lactating mothers in Depok City, Indonesia | 2 | 1 | 5 | 2 | 2 | 2 | 14 | high risk |
| Gibson, 2020, Indonesia (Bandung and Sumedang) | Association of maternal diet, micronutrient status, and milk volume with milk micronutrient concentrations in Indonesian mothers at 2 and 5 months postpartum | 2 | 1 | 1 | 1 | 2 | 2 | 9 | low risk |
| Hamid, 2019, Malaysia (Selangor) | Utilization of a Diet Optimization Model in Ensuring Adequate Intake among Pregnant Women in Selangor, Malaysia | 2 | 2 | 1 | 1 | 2 | 2 | 10 | moderate risk |
| Hartini, 2003, Indonesia (Purworejo) | Nutrient intake and iron status of urban poor and rural poor without access to rice fields are affected by the emerging economic crisis: the case of pregnant Indonesian women | 2 | 2 | 2 | 1 | 2 | 1 | 10 | Moderate risk |
| Hartriyanti, 2012, Indonesia | Nutrient intake of pregnant women in Indonesia: a review | 2 | 2 | 2 | 2 | 2 | 1 | 11 | Moderate risk |
| Hasbullah, 2019, Malaysia | Factors associated with dietary glycemic index and glycemic load in pregnant women and risk for gestational diabetes mellitus |  |  |  |  |  |  | 0 |  |
| Hassan, 2020, Malaysia (Sepang) | The effectiveness of theory-based intervention to improve haemoglobin levels among women with anaemia in pregnancy | 2 | 1 | 1 | 1 | 2 | 2 | 9 | Low risk |
| Ilmiawati, 2020, Indonesia (West Sumatera) | Sunlight exposed body surface area is associated with serum 25-hydroxyvitamin D (25(OH)D) level in pregnant Minangkabau women, Indonesia | 2 | 1 | 2 | 1 | 2 | 2 | 10 | moderate risk |
| Kamaruzzaman, 2018, Malaysia (Kuantan, Pahang) | The association of maternal diet and polyamines in human milk: A study among malay ethnic mothers in kuantan, malaysia | 2 | 1 | 3 | 1 | 2 | 2 | 11 | moderate risk |
| Kardjati, 1988, Indonesia (Madura) | Energy supplementation in the last trimester of pregnancy in East Java: I. Effect on birthweight | 1 | 1 | 1 | 1 | 2 | 2 | 8 | low risk |
| Kardjati, 1990, Indonesia (Madura) | Energy supplementation in the last trimester of pregnancy in East Java, Indonesia: effect on maternal anthropometry | 1 | 1 | 1 | 1 | 2 | 2 | 8 | low risk |
| Khor, 2021, Malaysia (Kuala lumpur) | Temporal Changes in Breast Milk Fatty Acids Contents: A Case Study of Malay Breastfeeding Women | 2 | 1 | 2 | 1 | 2 | 2 | 10 | moderate risk |
| Kneebone, 1985, Malaysia (Penang) | Fatty acid composition of breast milk from three racial groups from Penang, Malaysia | 2 | 2 | 6 | 1 | 2 | 2 | 15 | high risk |
| Lee, 2020, Malaysia (Selangor) | Influence of vitamin D binding protein polymorphism, demographics and lifestyle factors on vitamin D status of healthy Malaysian pregnant women | 2 | 1 | 3 | 1 | 2 | 2 | 11 | moderate risk |
| Loy, 2011, Malaysia | Development, validity and reproducibility of a food frequency questionnaire in pregnancy for the Universiti Sains Malaysia birth cohort study | 2 | 1 | 3 | 1 | 2 | 2 | 10 | moderate risk |
| Loy, 2011, Malaysia | **Higher intake of fruits and vegetables in pregnancy is associated with birth size** | 2 | 1 | 3 | 1 | 1 | 2 | 10 | moderate risk |
| Loy, 2013, Malaysia | Association between maternal food group intake and birth size | 2 | 1 | 3 | 1 | 1 | 2 | 10 | moderate risk |
| Madanijah, 2016, Indonesia (Bogor) | Nutritional status of lactating women in Bogor district, Indonesia: cross-sectional dietary intake in three economic quintiles and comparison with pre-pregnant women | 2 | 1 | 2 | 1 | 1 | 2 | 9 | low risk |
| Madanijah, 2016, Indonesia (Bogor) | Nutritional status of pre-pregnant and pregnant women residing in Bogor district, Indonesia: a cross-sectional dietary and nutrient intake study | 2 | 1 | 2 | 1 | 1 | 2 | 9 | low risk |
| Mahdy, 2014, Malaysia (Kuala Lumpur) | Antenatal calcium intake in Malaysia | 2 | 2 | 2 | 1 | 1 | 2 | 10 | moderate risk |
| Marsubrin, 2021, Indonesia | Preterm human milk composition and dietary intake of breastfeeding mothers in the Indonesian population | 2 | 1 | 2 | 1 | 2 | 2 | 10 | moderate risk |
| Mohamed, 2014, Malaysia (Kota Bharu) | Maternal Serum and Breast Milk Vitamin D Levels: Findings from the Universiti Sains Malaysia Pregnancy Cohort Study | 2 | 1 | 2 | 1 | 2 | 2 | 10 | moderate risk |
| Mutalazimah, 2015, Indonesia (Boyolali) | Energy, protein intake and mid-upper arm circumference in pregnant women in boyolali regency, Indonesia | 1 | 1 | 3 | 1 | 2 | 2 | 10 | moderate risk |
| Nadimin, 2019, Indonesia (Makassar) | Increasing of nutrition status of pregnant women after supplementation of moringa leaf extract (Moringa oliefera) in the coastal area of Makassar, Indonesia | 1 | 2 | 5 | 2 | 2 | 2 | 14 | high risk |
| Nahrisah, 2019, Indonesia (Aceh) | Micronutrient Intake And Perceived Barriers Among Anaemic Pregnant Women In Aceh, Indonesia | 2 | 1 | 2 | 1 | 2 | 2 | 10 | moderate risk |
| Nahrisah, 2020, Indonesia (Aceh) | Effect of Integrated Pictorial Handbook Education and Counseling on Improving Anemia Status, Knowledge, Food Intake, and Iron Tablet Compliance Among Anemic Pregnant Women in Indonesia: A Quasi-Experimental Study | 1 | 2 | 2 | 1 | 2 | 1 | 9 | low risk |
| Pee, 1995, Indonesia (Bogor) | Lack of improvement in vitamin A status with increased consumption of dark-green leafy vegetables | 1 | 2 | 5 | 2 | 2 | 2 | 14 | high risk |
| Persson, 2001, Indonesia (Purworejo) | Variability in nutrient intakes among pregnant women in Indonesia: implications for the design of epidemiological studies using the 24-H dietary recall method | 1 | 1 | 2 | 1 | 2 | 2 | 9 | low risk |
| Persson, 2002, Indonesia (Purworejo) | Vitamin A intake is low among pregnant women in central Java, Indonesia | 1 | 1 | 2 | 1 | 2 | 2 | 9 | low risk |
| Rahmannia, 2019, Indonesia (Sumedang) | Poor dietary diversity and low adequacy of micronutrient intakes among rural Indonesian lactating women from Sumedang district, West Java | 1 | 1 | 2 | 1 | 2 | 2 | 9 | low risk |
| Savitri, 2018, Indonesia (Jakarta) | Ramadan during pregnancy and birth weight of newborns | 2 | 1 | 5 | 2 | 2 | 2 | 14 | high risk |
| Sulchan, 2016, Indonesia | Dietary plant food and socioeconomic determinants of vitamin A status : study in rural lactating woman during crisis in Central Java | 1 | 1 | 5 | 2 | 2 | 2 | 13 | moderate risk |
| Suprapto, 2002, Indonesia (Karanganyar) | Effect of low-dosage vitamin A and riboflavin on iron-folate supplementation in anaemic pregnant women | 1 | 1 | 5 | 2 | 2 | 2 | 13 | moderate risk |
| Sutrisna, 2018, Indonesia | Iodine Intake Estimation from the Consumption of Instant Noodles, Drinking Water and Household Salt in Indonesia | 1 | 2 | 5 | 1 | 2 | 1 | 12 | moderate risk |
| Tan, 2020, Malaysia (Kajang and Seng) | Case study of temporal changes in maternal dietary intake and the association with breast milk mineral contents | 2 | 1 | 5 | 2 | 2 | 2 | 14 | high risk |
| Tan, 2020, Malaysia (Selangor and Kuala lumpur) | Associations of obstetrical characteristics and dietary intakes with iron status among pregnant women in Selangor and Kuala Lumpur | 1 | 1 | 3 | 1 | 1 | 2 | 9 | low risk |
| Wibowo, 2016, Indonesia (Jakarta) | Effects of Bifidobacterium animalis lactis HN019 (DR10TM), inulin, and micronutrient fortified milk on faecal DR10TM, immune markers, and maternal micronutrients among Indonesian pregnant women | 1 | 1 | 3 | 1 | 2 | 2 | 10 | moderate risk |
| Woon, 2019, Malaysia (Selangor and Kuala lumpur) | Vitamin D deficiency during pregnancy and its associated factors among third trimester Malaysian pregnant women | 1 | 1 | 3 | 1 | 2 | 2 | 10 | moderate risk |
| Yeop, 2018, Malaysia | Hypocalcaemia and its contributing factors among first trimester pregnant women in an urban area in Malaysia | 1 | 1 | 3 | 1 | 2 | 2 | 10 | moderate risk |
| Yong, 2017, Malaysia | Pre-pregnancy BMI and intake of energy and calcium are associated with the vitamin D intake of pregnant Malaysian women | 2 | 1 | 2 | 1 | 2 | 2 | 10 | moderate risk |
| Yusrawati, 2017, Indonesia | Analyses of Nutrients and Body Mass Index as Risk Factor for Preeclampsia | 1 | 1 | 6 | 2 | 2 | 2 | 14 | high risk |
| Zaleha, 2015, Malaysia (Selangor) | Development and validation of a food frequency questionnaire for vitamin D intake among urban pregnant women in Malaysia | 2 | 1 | 1 | 1 | 1 | 2 | 8 | low risk |
