# Supplementary figures and images for: Nutrient intakes of pregnant and lactating women in Indonesia and Malaysia: Systematic review and meta-analysis
Source: Front Nutr. 2023 Mar 30;10:1030343. doi: 10.3389/fnut.2023.1030343 (PMC10098007; doi:10.3389/fnut.2023.1030343)

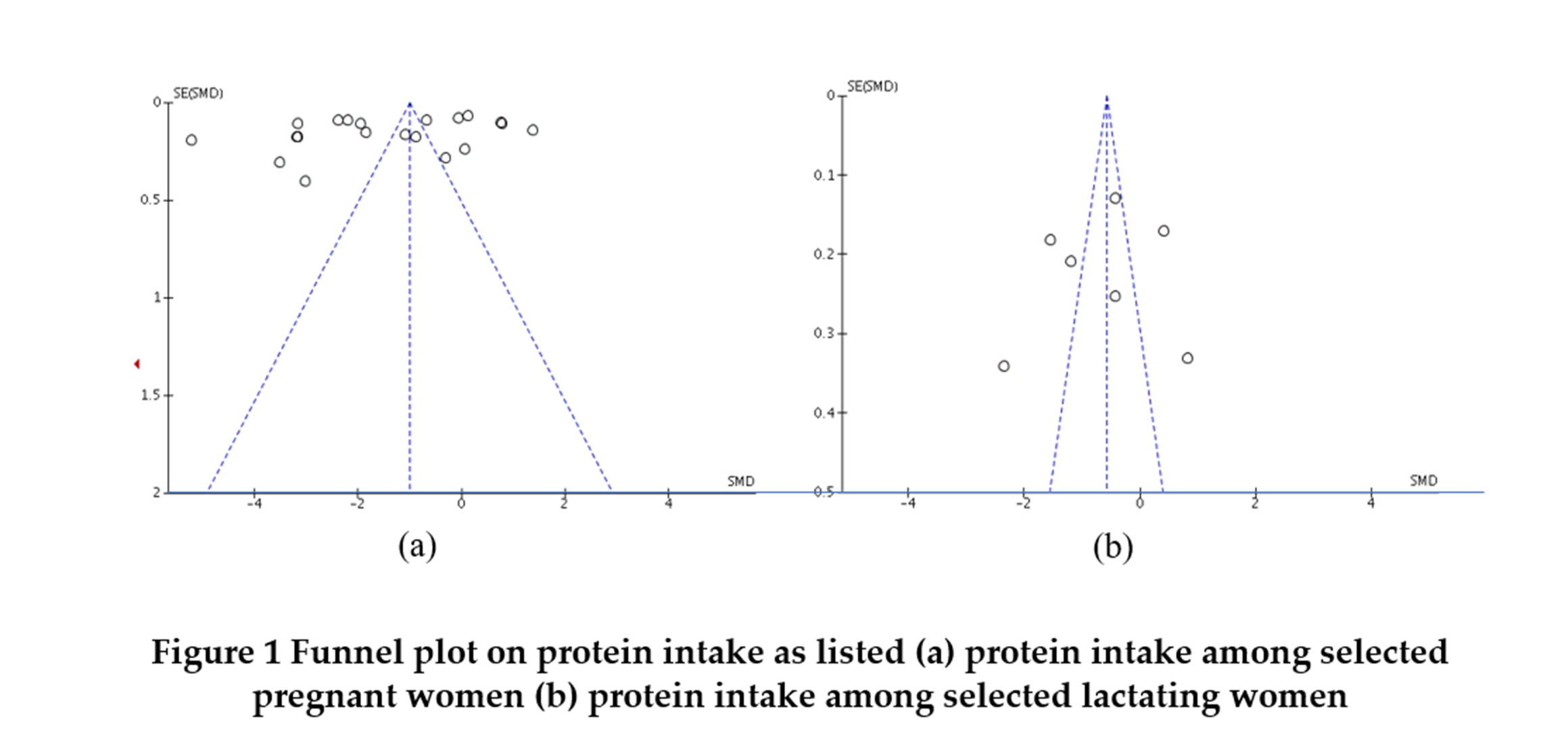

Supplement: Supplementary file 3 [file Image_1.tif]

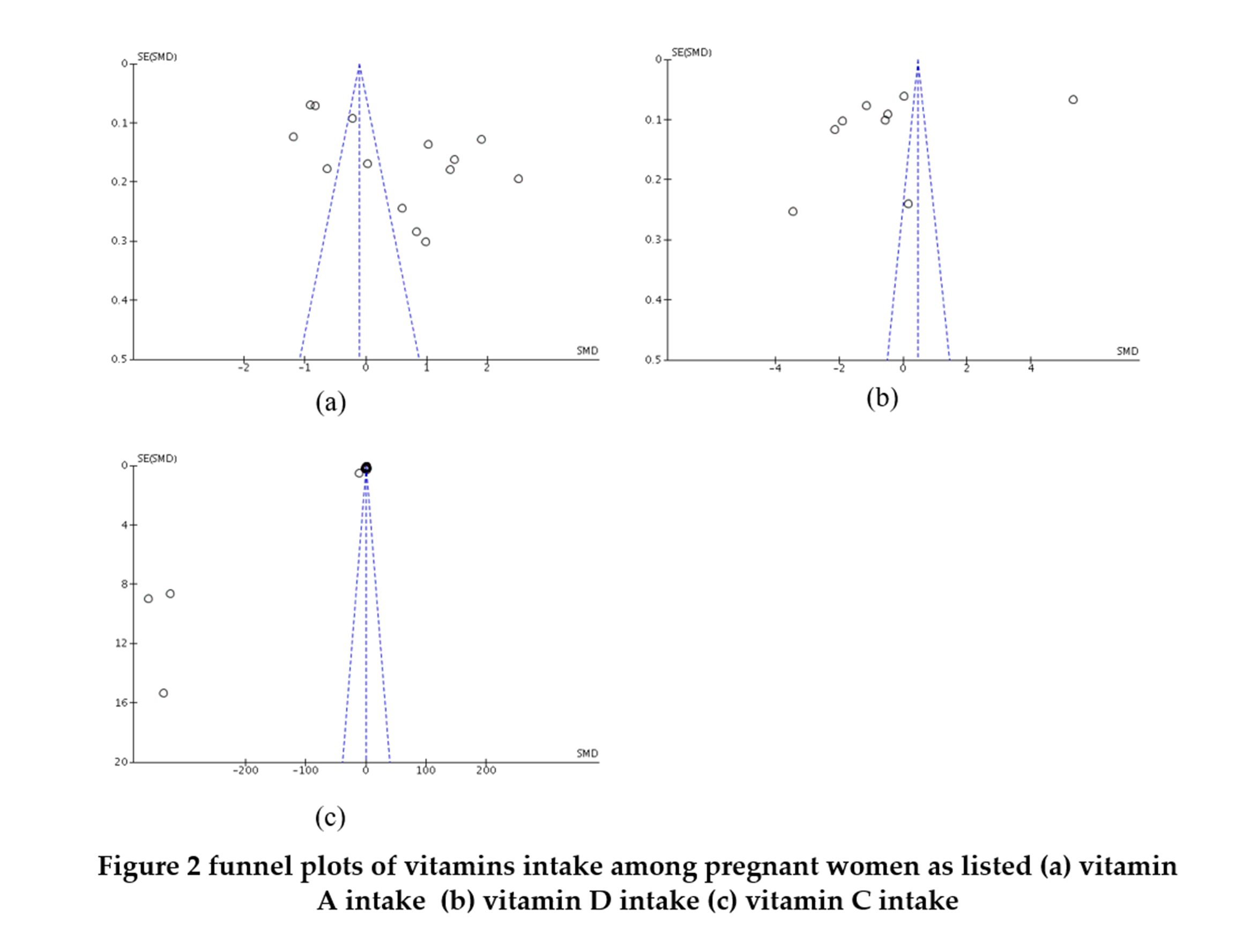

Supplement: Supplementary file 4 [file Image_2.tif]

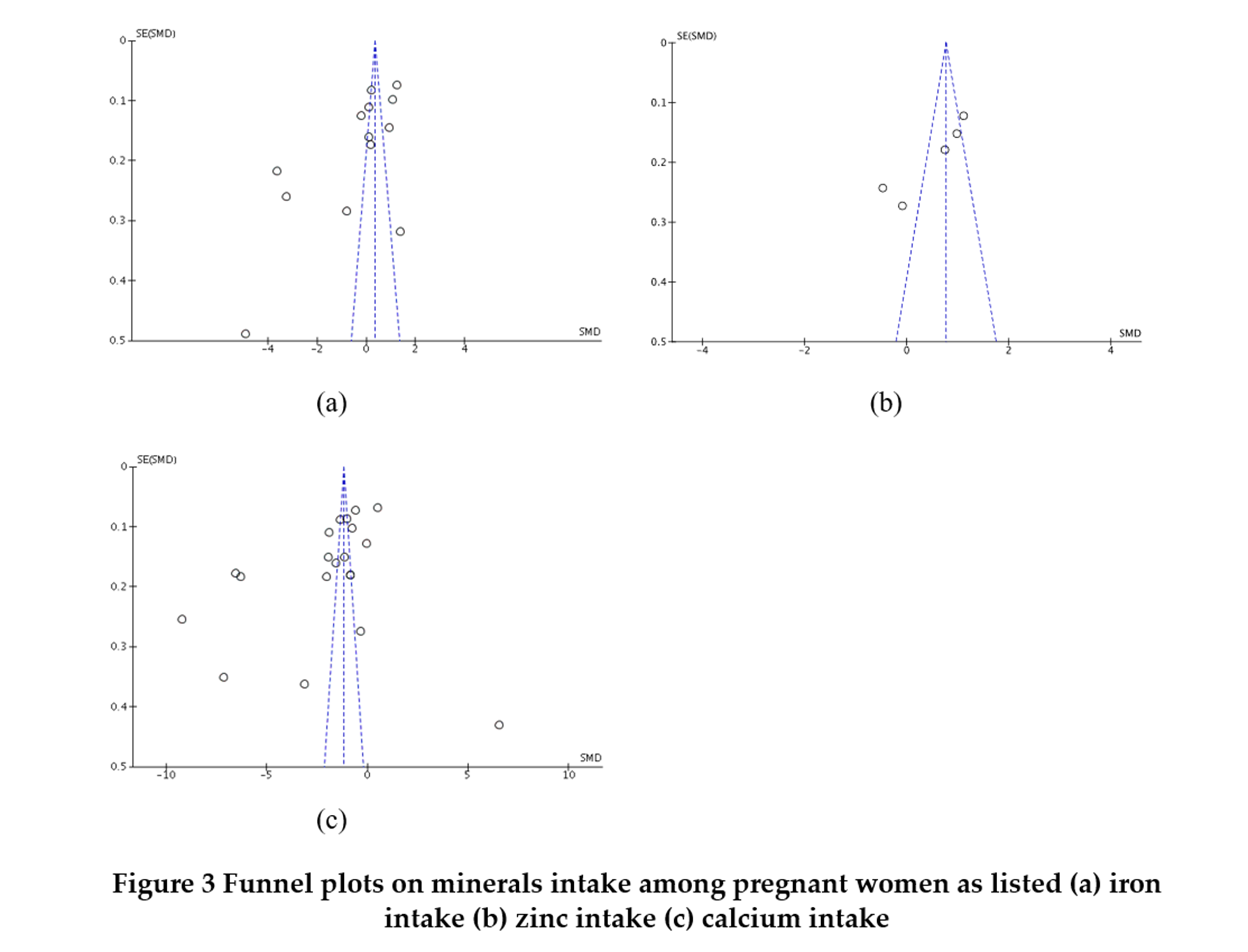

Supplement: Supplementary file 5 [file Image_3.tif]

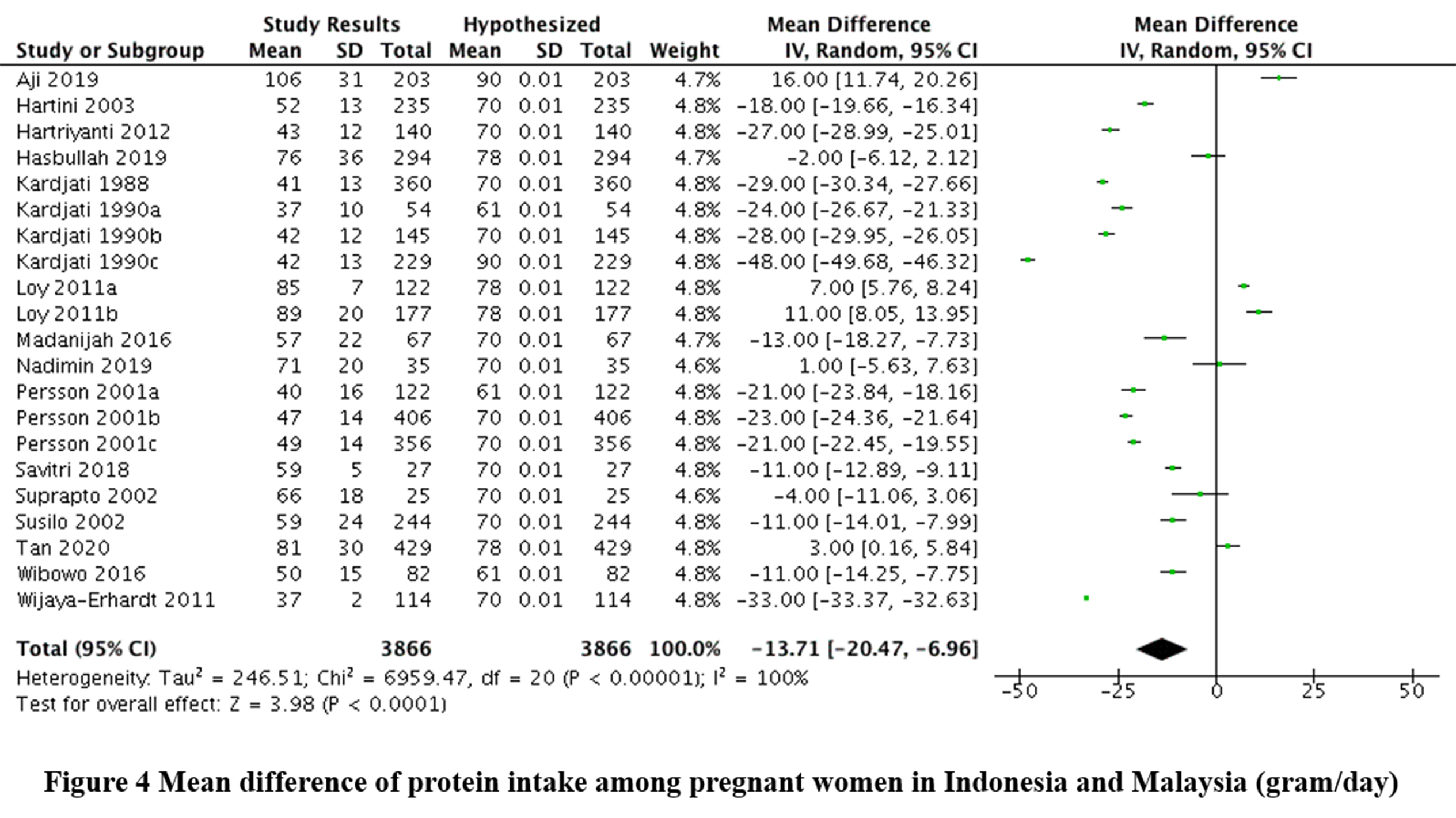

Supplement: Supplementary file 6 [file Image_4.tif]

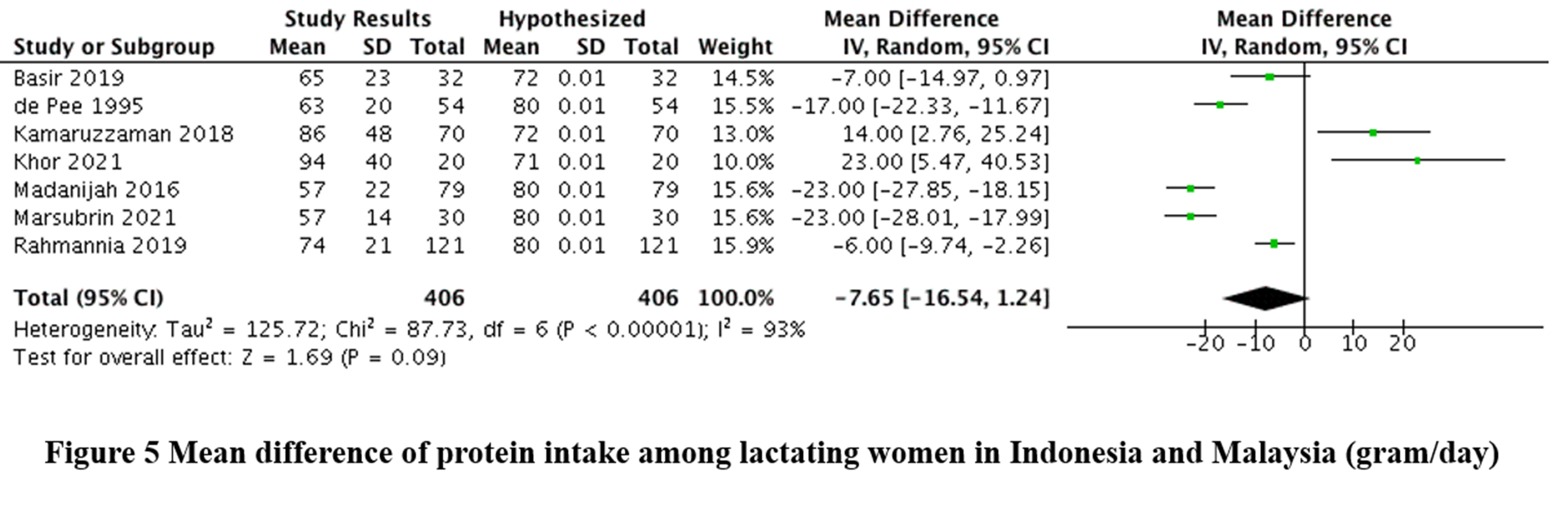

Supplement: Supplementary file 7 [file Image_5.tif]

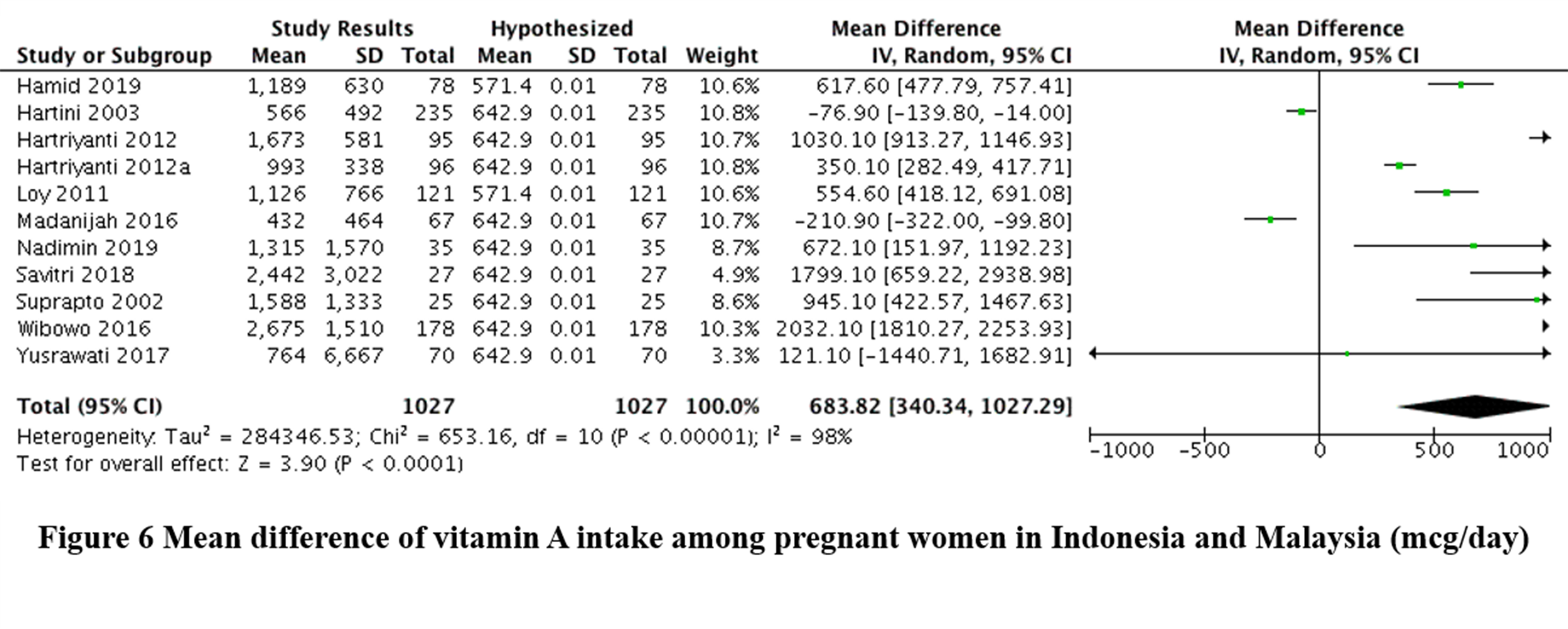

Supplement: Supplementary file 8 [file Image_6.tif]

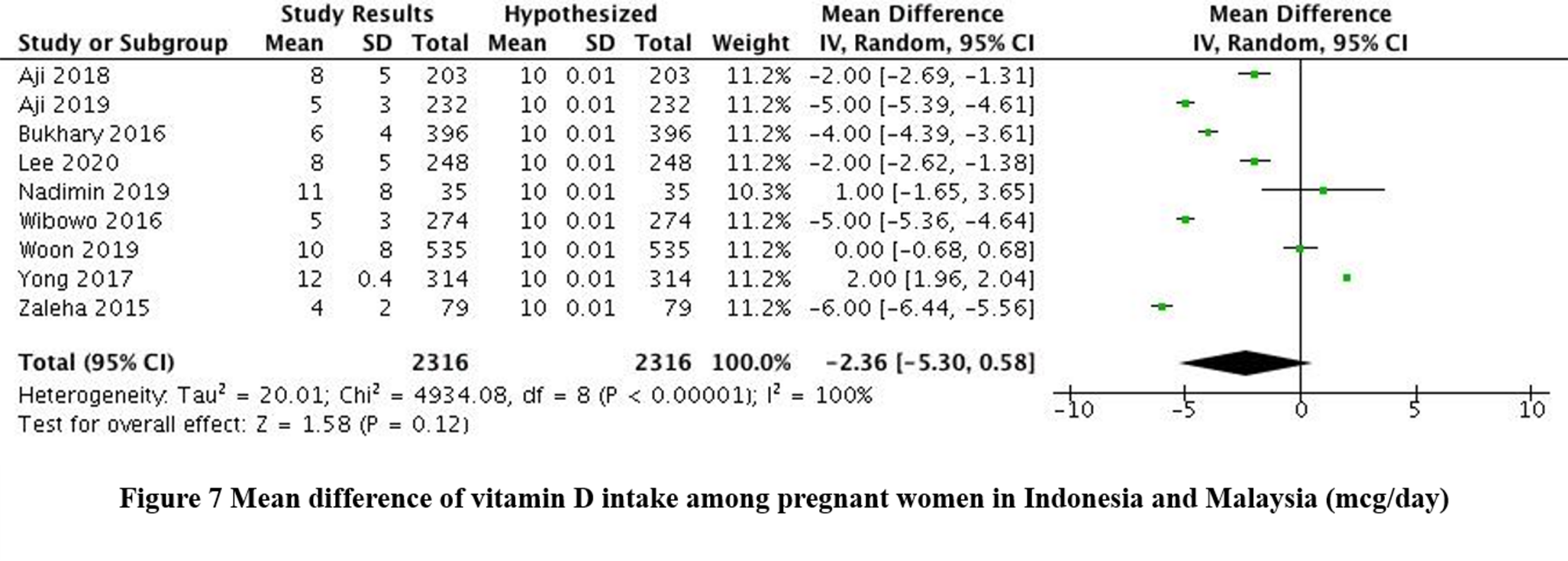

Supplement: Supplementary file 9 [file Image_7.tif]

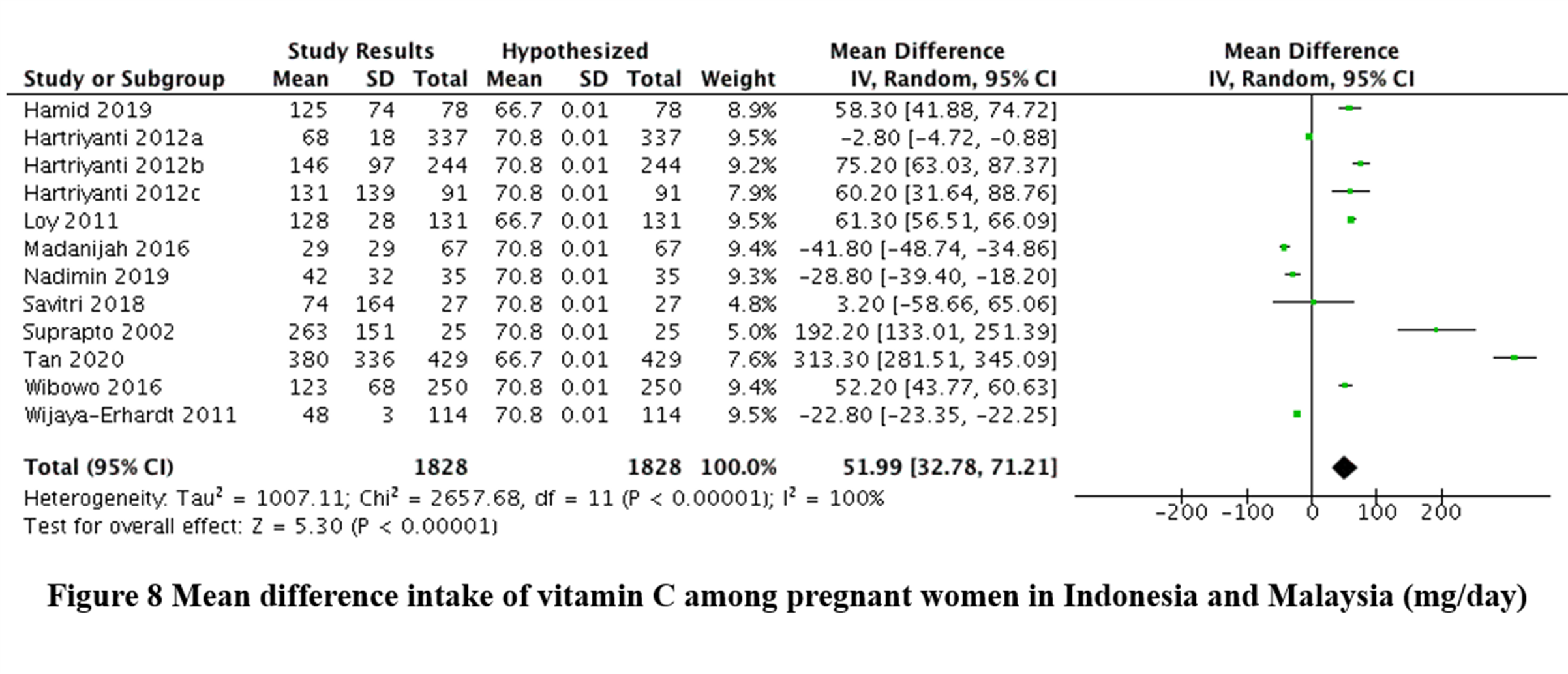

Supplement: Supplementary file 10 [file Image_8.tif]

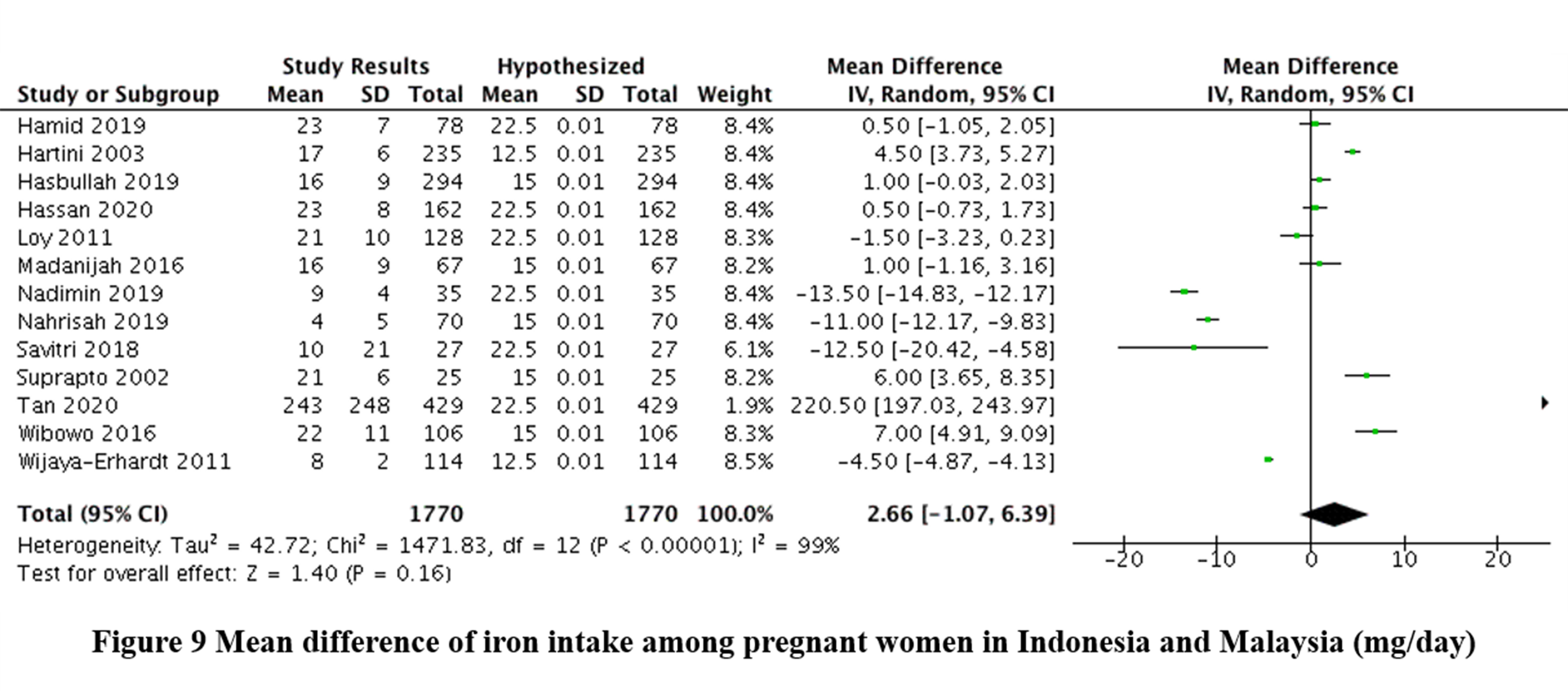

Supplement: Supplementary file 11 [file Image_9.tif]

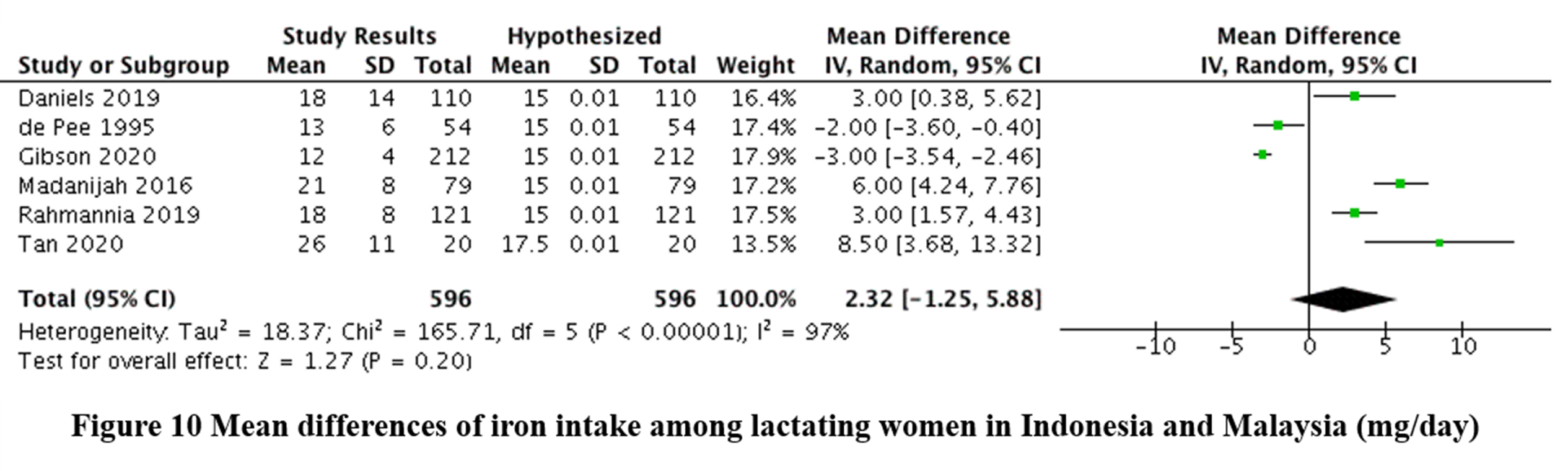

Supplement: Supplementary file 12 [file Image_10.tif]

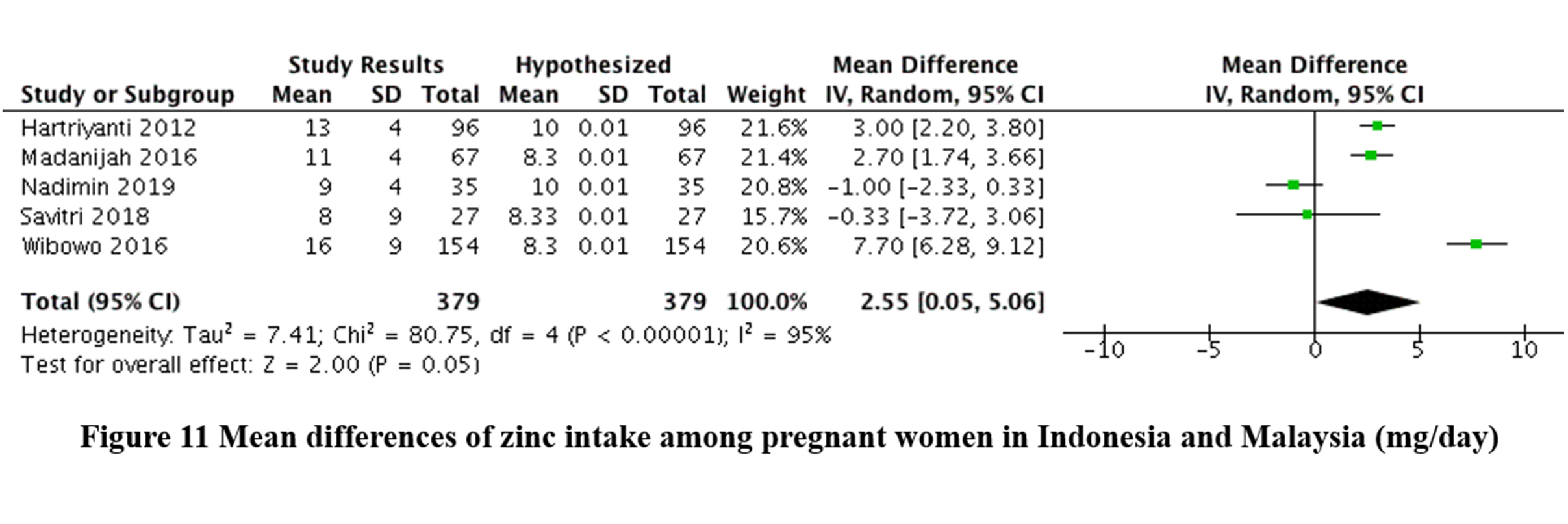

Supplement: Supplementary file 13 [file Image_11.tif]

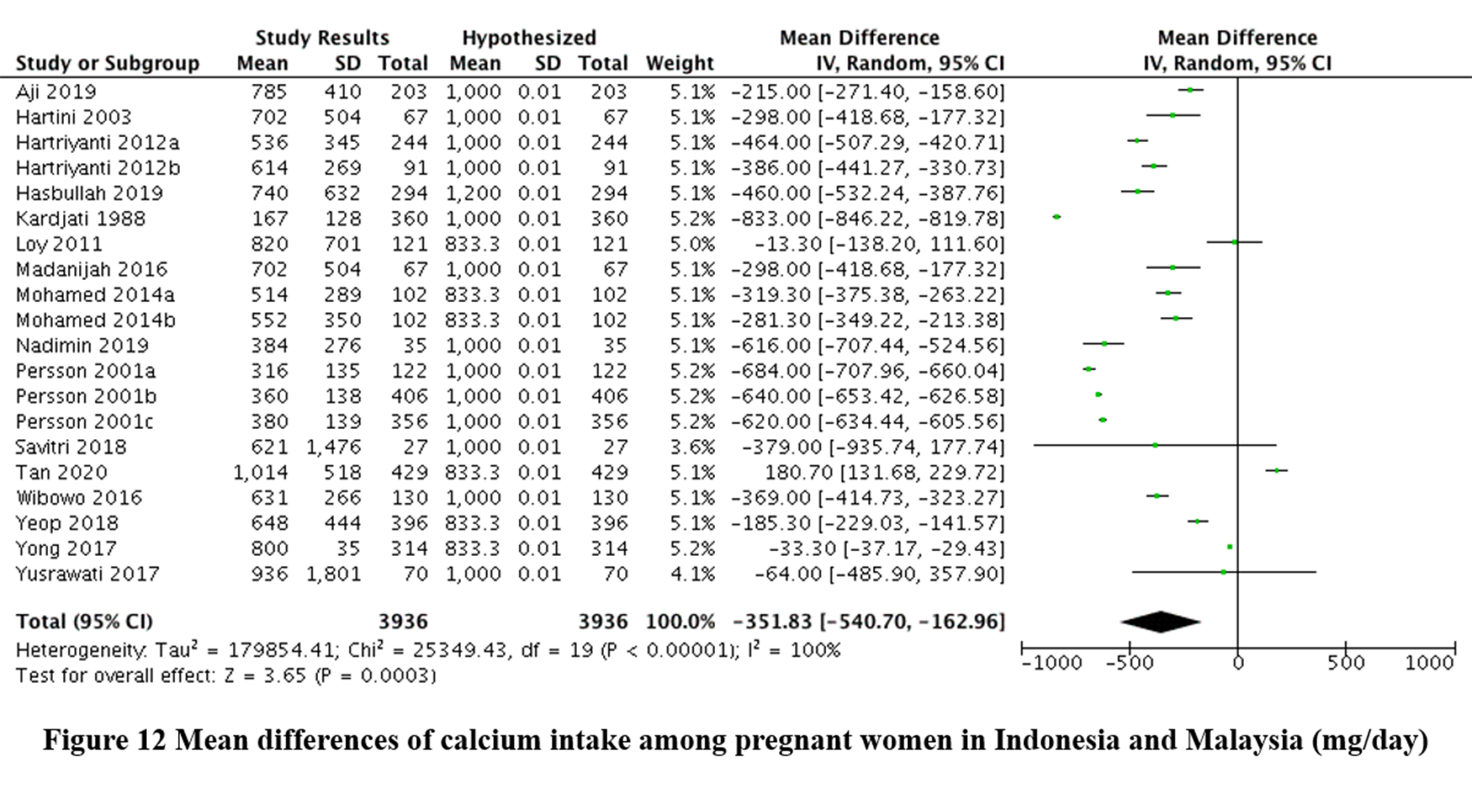

Supplement: Supplementary file 14 [file Image_12.tif]
